# National Trends in American Heart Association Revised Life's Simple 7 Metrics Associated With Risk of Mortality Among US Adults

**DOI:** 10.1001/jamanetworkopen.2019.13131

**Published:** 2019-10-11

**Authors:** Liyuan Han, Dingyun You, Wenjie Ma, Thomas Astell-Burt, Xiaoqi Feng, Shiwei Duan, Lu Qi

**Affiliations:** 1 Zhejiang Provincial Key Laboratory of Pathophysiology, School of Medicine, Department of Epidemiology, Ningbo University, Ningbo, China; 2 Department of Epidemiology, School of Public Health and Tropical Medicine, Tulane University, New Orleans, Louisiana; 3 School of Public Health, Kunmming Medical University, Kunming, China; 4 Clinical and Translational Epidemiology Unit, Division of Gastroenterology, Massachusetts General Hospital, Boston; 5 Population Wellbeing and Environment Research Lab, School of Health and Society, Faculty of Social Sciences, University of Wollongong, Wollongong, Australia; 6 Menzies Centre for Health Policy, University of Sydney, Sydney, Australia; 7 llawarra Health and Medical Research Institute, University of Wollongong, Wollongong, Australia; 8 School of Public Health, Peking Union Medical College, The Chinese Academy of Medical Sciences, Beijing, China; 9 Department of Nutrition, Harvard T.H. Chan School of Public Health, Boston, Massachusetts; 10 Channing Division of Network Medicine, Department of Medicine, Brigham and Women’s Hospital, Harvard Medical School, Boston, Massachusetts

## Abstract

**Importance:**

The American Heart Association (AHA) introduced the Life’s Simple 7 (LS7) metrics to assess and promote cardiovascular health. However, several shortcomings of these metrics have been identified. Therefore, a revised set of LS7 metrics was developed.

**Objectives:**

To evaluate national trends in the metrics addressed by the revised LS7 and the individual and combined associations of the revised LS7 metrics with all-cause and cause-specific mortality and to compare these measures with the AHA recommended LS7 metrics.

**Design, Setting, and Participants:**

This national cross-sectional study used data from the National Health and Nutrition Examination Survey from 1988 to 2016. The revised LS7 metrics included a combination of the body mass index and waist to hip ratio, Healthy Eating Index–2010, and a lower blood pressure threshold of greater than or equal to 130/80 mm Hg in addition to physical activity, smoking, total cholesterol, and fasting blood glucose. Data for this study were analyzed from June 1, 2017, to December 31, 2017.

**Main Outcomes and Measures:**

The primary outcome was all-cause mortality. The secondary outcome was cancer and cardiovascular disease (CVD) mortality.

**Results:**

Data were available for 13 606 adults in 1988 to 1994 (7329 [53%] female; mean [SD] age, 47 [17.7] years), 6360 in 1999 to 2004 (3442 [54%] female; mean [SD] age, 47 [18.6] years), 10 618 in 2005 to 2010 (5428 [51%] female; mean [SD] age, 47 [17.5] years), and 10 773 in 2011 to 2016 (5474 [50%] female; mean [SD] age, 48 [17.4] years). Compared with a revised LS7 score of 0 to 1, the adjusted hazard ratios for a revised LS7 score of 5 to 7 were 0.46 (95% CI, 0.35-0.61) for all-cause mortality, 0.42 (95% CI, 0.25-0.68) for cancer mortality, and 0.37 (95% CI, 0.24-0.55) for CVD mortality, respectively. The adjusted hazard ratios for participants who met 6 or more AHA recommended ideal LS7 metrics were 0.49 (95% CI, 0.33-0.74) for all-cause mortality, 0.60 (95% CI, 0.29-1.25) for cancer mortality, and 0.24 (95% CI, 0.13-0.47) for CVD mortality. Participants with a body mass index of 29.9 or less but without central obesity were independently associated with lower risk of all-cause and CVD mortality. Blood pressure was associated with 36.7% or more of the observed population-attributable fraction of mortality.

**Conclusions and Relevance:**

The individual revised LS7 metrics with modified criteria regarding weight, blood pressure, and diet provide more information about factors associated with cancer mortality than the original AHA LS7 metrics.

## Key Points

**Question:**

Are the revised Life’s Simple 7 (LS7) metrics associated with reduced risk of all-cause, cancer, and cardiovascular disease mortality compared with the original American Heart Association LS7 metrics?

**Findings:**

This cross-sectional study found that, compared with the original metrics, the revised LS7 metrics were associated with reduced cancer mortality. For participants with body mass index less than or equal to 29.9 but without central obesity, the revised metrics were independently associated with decreased risk of all-cause and cardiovascular disease mortality, with blood pressure having the greatest association with all mortality outcomes.

**Meaning:**

These findings indicate that modified criteria regarding weight, blood pressure, and diet in the revised LS7 provide additional information about factors associated with cancer mortality.

## Teaser

This cross-sectional study examines the association of adherence to the American Heart Association (AHA) revised Life’s Simple 7 (LS7) metrics with reduced risk of all-cause, cancer, and cardiovascular disease–related mortality and compares the revised metrics with the original LS7 metrics.

## Introduction

The American Heart Association (AHA) proposed the Life’s Simple 7 (LS7) set of risk factors used to indicate cardiovascular health.^1^ The LS7 comprises 7 ideal metrics, including 3 ideal health factors: untreated systolic blood pressure (BP) less than 120 and diastolic BP less than 80 mm Hg, untreated total cholesterol level less than 200 mg/dL (to convert to millimoles per liter, multiply by 0.0259), and untreated fasting blood glucose concentration less than 100 mg/dL (to convert to millimoles per liter, multiply by 0.0555). The LS7 also includes 4 ideal health behaviors: not smoking, maintaining a body mass index (BMI; calculated as weight in kilograms divided by height in meters squared) less than 25, achievement of a goal physical activity level, and a diet meeting 4 to 5 target components (recommended consumption levels of fruits and vegetables, fish, fiber-rich whole grains, sodium, and sugar-sweetened beverages).^1^ In previous studies, adherence to the ideal LS7 metrics was found to be associated with decreased risks of cardiovascular disease (CVD),^2^ all-cause mortality,^3^ and cancer.^4^

However, further consideration suggests that a reconstruction of the LS7 metrics should include additional factors. For example, although the AHA-proposed LS7 metrics include a BMI of less than 25 (a commonly used surrogate for normal weight),^1^ this measure is prone to misclassification, especially in older populations.^5,6^ Central obesity, which is defined by the waist to hip ratio (WHR), is a more sensitive marker of body fat distribution that is associated with higher mortality independent of BMI.^2^ Furthermore, the 2015 to 2020 Dietary Guidelines for Americans,^7^ which provide updated evidence relevant to reducing the cardiovascular risk and additional recommendations for adopting a healthy diet and lifestyle, recommend an integrated dietary pattern (Healthy Eating Index–2010). Moreover, the new 2017 American College of Cardiology/American Heart Association guideline has defined a new threshold for hypertension as a BP greater than or equal to 130/80 mm Hg.^8^ Accordingly, we included modified weight, diet, and BP criteria in a revised set of LS7 metrics with an intention to more efficiently assess cardiovascular health in populations.

We used nationally representative data from the US National Health and Nutrition Examination Surveys (NHANES) from 1988 to 2016 to estimate national trends in the revised LS7 metrics among US adults aged 20 years and older. We also evaluated the individual and combined associations of the revised LS7 metrics on all-cause and cause-specific (eg, cancer and CVD) mortality and estimated the population-attributable fractions (PAF) associated with adherence to each ideal revised LS7 metric and the combination thereof. The comparison with the AHA-recommended LS7 metrics was also investigated.

## Methods

### Study Population

The NHANES is an ongoing, multistage, cross-sectional survey. Two data sets were used: NHANES III, conducted from 1988 to 1994,^8^ and the continuous NHANES survey, with data collected from 1999 to 2016. The National Center for Health Statistics Research Ethics Review Board reviewed and approved NHANES, and informed consent was obtained for all participants. This survey uses a complex, stratified, multistage probability sampling design to deliver nationally representative data on the health and nutritional status of the noninstitutionalized civilian population across the United States. Specifically, the NHANES obtains abundant information about a range of health topics through in-home interviews that address demographic, socioeconomic, dietary, and health-related questions, followed by blood sampling at a mobile examination center. The Medical School of Ningbo University Review Board determined that the current study was exempt from review and patient informed consent given the use of publicly available data. This study followed the Strengthening the Reporting of Observational Studies in Epidemiology (STROBE) guideline for cross-sectional studies.

Our study is based on an analysis of data from the survey cycles corresponding to NHANES III (1988-1994), 1999 to 2004, 2005 to 2010, and 2011 to 2016. Participants with a BMI less than 18.5 or a history of heart attack, congestive heart failure, stroke, skin cancer, and other cancers were excluded because these factors are associated with a higher risk of mortality.^9,10^ Participants who were pregnant or who were younger than 20 years were also excluded.

### Definitions of the Revised LS7 Metrics

#### Smoking Status

Current smokers were defined as those who answered yes to the following questions: “Do you smoke cigarettes now?” and “Have you smoked at least 100 cigarettes in your lifetime?” Former smokers included those who responded no to the first question but yes to the second. Never smokers were those who responded no to both questions. The status of never smokers, former smokers, and current smokers was defined as ideal, intermediate, and poor, respectively (Table 1).

**Table 1.** Definitions of Ideal, Intermediate, and Poor LS7 Metrics for Adults

Goal/Metric	AHA Definitions of LS7 Metrics^a^	Definitions for Revised LS7 Metrics			
		NHANES III, 1988-1994	NHANES 1999-2004	NHANES 2005-2010	NHANES 2011-2016
**Smoking Status**					
Ideal	Never or quit >12 mo ago	Survey did not ask the time since quitting smoking; we included the participants who self-reported never smoking in the ideal category for consistent estimates across the surveys	Survey asked the time since quitting smoking; to be consistent with NHANES III estimate, we included the participants who self-reported never smoking in the ideal category	Survey asked the time since quitting smoking; to be consistent with NHANES III estimate, we included the participants who self-reported never smoking in the ideal category	Survey asked the time since quitting smoking; to be consistent with NHANES III estimate, we included the participants who self-reported never smoking in the ideal category
Intermediate	Former or quit ≤12 mo	Included all former smokers	Included all former smokers	Included all former smokers	Included all former smokers
Poor	Current smoking	Current smoking	Current smoking	Current smoking	Current smoking
**BMI-WHR**^b^					
1	BMI <25	BMI ≤24.9 and WHR <0.9 for men; BMI ≤24.9 and WHR<0.8 for women	NA	NA	NA
2	BMI 25-29.9	BMI ≤24.9 and WHR <1 for men; BMI ≤24.9 and WHR <0.92 for women	NA	NA	NA
3	BMI ≥30.0	BMI ≤24.9 and WHR >1 for men; BMI ≤24.9 and WHR >0.92 for women	NA	NA	NA
4		BMI ≤29.9 and WHR<0.9 for men; BMI ≤29.9 and WHR<0.8 for women	NA	NA	NA
5		BMI ≤29.9 and WHR <1 for men; BMI ≤29.9 and WHR <0.92 for women	NA	NA	NA
6		BMI ≤29.9 and WHR >1 for men; BMI ≤29.9 and WHR >0.92 for women	NA	NA	NA
7		BMI ≥30 and WHR <0.9 for men; BMI ≥30 and WHR <0.8 for women	NA	NA	NA
8		BMI ≥30 and WHR <1 for men; BMI ≥30 and WHR <0.92 for women	NA	NA	NA
9		BMI ≥30 and WHR >1 for men; BMI ≥30 and WHR >0.92 for women	NA	NA	NA
**Physical Activity**					
Ideal	≥150 min/wk moderate or ≥75 min/wk vigorous or ≥150 min/wk moderate + vigorous	NHANES III did not ask the duration of physical activities; we defined physically active as engaging in physical activities with 3≤METS<6 and ≥5 times/wk or physical activities with METS ≥6 and 3.0 times/wk. Physical activities included walking, jogging or running, bicycling, swimming, aerobics or aerobic dancing, other dancing, calisthenics, gardening or yard work, and other sports	≥150 min/wk moderate or ≥75 min/wk vigorous or ≥150 min/wk moderate + vigorous; physical activities included walking, jogging or running, bicycling, swimming, aerobics or aerobic dancing, other dancing, calisthenics, gardening or yard work, and other sports	≥150 min/wk moderate or ≥75 min/wk vigorous or ≥150 min/wk moderate + vigorous; physical activities included walking, jogging or running, bicycling, swimming, aerobics or aerobic dancing, other dancing, calisthenics, gardening or yard work, and other sports	≥150 min/wk moderate or ≥75 min/wk vigorous or ≥150 min/wk moderate + vigorous; physical activities included walking, jogging or running, bicycling, swimming, aerobics or aerobic dancing, other dancing, calisthenics, gardening or yard work, and other sports
Intermediate	1-149 min/wk moderate or 1-74 min/wk vigorous or 1-149 min/wk moderate + vigorous	The difference between physically active and no physical activity was taken as intermediate	1-149 min/wk moderate or 1-74 min/wk vigorous or 1-149 min/wk moderate + vigorous	1-149 min/wk moderate or 1-74 min/wk vigorous or 1-149 min/wk moderate + vigorous	1-149 min/wk moderate or 1-74 min/wk vigorous or 1-149 min/wk moderate + vigorous
Poor	None	None	None	None	None
**Healthy Diet Score**^c^					
Ideal	4-5 Components^d^	Healthy eating index score ≥69.3	Healthy eating index score ≥69.3	Healthy eating index score ≥69.3	Healthy eating index score ≥69.3
Intermediate	2-3 Components^d^	Healthy eating index score 56.9-69.2	Healthy eating index score 56.9-69.2	Healthy eating index score 56.9-69.2	Healthy eating index score 56.9-69.2
Poor	0-1 Components^d^	Healthy eating index score <56.9	Healthy eating index score <56.9	Healthy eating index score <56.9	Healthy eating index score <56.9
**Total Cholesterol**					
Ideal	<200 mg/dL	<200 mg/dL	<200 mg/dL	<200 mg/dL	<200 mg/dL
Intermediate	200-239 mg/dL or treated to goal	200-239 mg/dL or treated to goal	200-239 mg/dL or treated to goal	200-239 mg/dL or treated to goal	200-239 mg/dL or treated to goal
Poor	≥240 mg/dL	≥240 mg/dL	≥240 mg/dL	≥240 mg/dL	≥240 mg/dL
**Blood Pressure**					
Ideal^e^	SBP <120/DBP <80 mm Hg	SBP <120/DBP <80 mm Hg	SBP <120/DBP <80 mm Hg	SBP <120/DBP <80 mm Hg	SBP <120/DBP <80mmHg
Intermediate	SBP 120-139 or DBP 80-89 mm Hg or treated to goal	SBP 120-129 or DBP <80 mm Hg or treated to goal	SBP 120-129 or DBP <80 mm Hg or treated to goal	SBP 120-129 or DBP <80 mm Hg or treated to goal	SBP 120-129 or DBP <80 mm Hg or treated to goal
Poor	SBP ≥140 or DBP ≥90 mm Hg	SBP ≥130 or DBP ≥80 mm Hg	SBP ≥130 or DBP ≥80 mm Hg	SBP ≥130 or DBP ≥80 mm Hg	SBP ≥130 or DBP ≥80 mm Hg
**Fasting Plasma Glucose**					
Ideal	<100 mg/dL	<100 mg/dL; fasting glucose was available for a subsample of NHANES III participants (n = 6939)	<100 mg/dL; fasting glucose was available for a subsample of NHANES 1999-2004 participants (n = 5635)	<100 mg/dL; fasting glucose was available for a subsample of NHANES 2005-2010 participants (n = 4124)	<100 mg/dL; fasting glucose was available for a subsample of NHANES 2011-2014 participants (n = 6205)
Intermediate	100-125 mg/dL or treated to goal	100-125 mg/dL or treated to goal	100-125 mg/dL or treated to goal	100-125 mg/dL or treated to goal	100-125 mg/dL or treated to goal
Poor	≥126 mg/dL	≥126 mg/dL	≥126 mg/dL	≥126 mg/dL	≥126 mg/dL

Abbreviations: AHA, American Heart Association; BMI, body mass index (calculated as weight in kilograms divided by height in meters squared); DBP, diastolic blood pressure; LS7, Life’s Simple 7; METS, metabolic equivalent score; NA, not available; NHANES, National Health and Nutrition Examination Survey; SBP, systolic blood pressure; WHR, waist to hip ratio.

SI conversion factors: To convert cholesterol to mmol/L, multiply by 0.0259; fasting plasma glucose to mmol/L, multiply by 0.0555.

^a^ According to the AHA Strategic Impact Goal.^1^

^b^ The classification of BMI combined with WHR was based on the work of Sahakyan et al.^2^ Category 3 BMI-WHR (normal weight with central obesity) was defined as poor.

^c^ The healthy diet score was calculated based on the Healthy Eating Index–2010, a measure of diet quality comprising 12 components. Nine components assess dietary adequacy (intakes of total fruit, whole fruit, total vegetables, greens and beans, whole grains, dairy, total protein foods, and seafood and plant proteins and fatty acid ratio), with higher scores indicating higher consumption. Three components assess dietary components that should be consumed in moderation (refined grains, sodium, and empty calories), with higher scores indicating lower consumption.

^d^ The first dietary metrics include fruits and vegetables (≥4.5 cups per day), fish (2 or more 3.5-oz servings per week [preferably oily fish]), fiber-rich whole grains (3 or more 1-oz-equivalent servings per day), sodium (<1500 mg per day), and sugar-sweetened beverages (≤450 kcal [36 oz] per week). The secondary dietary metrics include nuts, legumes, and seeds (≥4 servings per week); processed meats (none or ≤2 servings per week); and saturated fat (<7% of total energy intake).

^e^ Untreated values, ie, no hypertension or high cholesterol levels.

#### Physical Activity

Prior to 1999, the NHANES III (1988-1994) assessed physical activity as the monthly frequency of participation in the following types of leisure-time physical activity: walking, jogging, biking, swimming, calisthenics, gardening, weight lifting, aerobics, dancing, and up to 4 additional activities. However, the NHANES III did not collect data about activity duration. Responses were standardized as times per week using the conversion factors 4.3 weeks per month and 30.4 days per month and rounded to the nearest whole number. Each activity was then assigned a metabolic equivalent score using a standardized coding scheme. Physical activity was calculated as the sum of the intensity rating multiplied by the times (of each activity) per week. For the NHANES III (1988-1994), ideal physical activity (physically active) was defined using the following cutoffs: a metabolic equivalent score of 3.0 to 5.9 and participation 5 or more times per week, or a metabolic equivalent score of 6.0 or greater and participation 3 or more times per week (Table 1).^11,12^

After 1999, participants were categorized into ideal, intermediate, or poor leisure-time physical activity levels based on whether they met the following recommendations for weekly activity: ideal, 75 minutes or more of vigorous activity or 150 minutes or more of moderate activity or 150 minutes or more of combined moderate and vigorous physical activity; intermediate, more than 0 minutes of physical activity but less than recommendations; and poor, 0 minutes of physical activity (Table 1).

#### Diet

The NHANES 1988 to 2016 estimated the Healthy Eating Index–2010 from 2 nonconsecutive 24-hour recall periods.^13^ The total healthy diet score ranged from 0 to 100, with greater scores indicating superior diet quality. This measure was grouped into tertiles: ideal, greater than 69.3; intermediate, 56.9 to 69.3; and poor, less than 56.9 (Table 1).

#### BMI and WHR

We used a sex-stratified composite classification of the BMI and WHR to account for sex-stratified differences within the same cohort.^14^ The detailed classifications are shown in eTable 1 in the Supplement. A BMI-WHR level of 3 or greater (normal weight with central obesity) was defined as poor; all other levels were defined as ideal.^14^ The NHANES 1999 to 2016 did not measure hip circumference, and therefore the WHR is not available for the NHANES 1999 to 2004, 2005 to 2010, and 2011 to 2016. Different combinations of the WHR and BMI were only available for NHANES III (1988-1994) (Table 1).

#### Hypertension

The new 2017 American College of Cardiology/American Heart Association guideline updated the threshold for hypertension to a BP of 130/80 mm Hg or greater.^8^ An untreated BP less than 120/80 mm Hg was defined as ideal (Table 1).

#### Cholesterol

An untreated total cholesterol level less than 200 mg/dL was defined as ideal. This value was identical to the AHA definition of ideal total cholesterol (Table 1).

#### Fasting Plasma Glucose

Hemoglobin A_1c_ values of less than 5.7% and less than 6.0% (to convert to proportion of total hemoglobin, multiply by 0.01) were used as proxies for fasting glucose levels less than 100 and less than 125 mg/dL, respectively. An untreated fasting blood glucose level of less than 100 mg/dL was defined as ideal (Table 1).

### Revised Combined LS7 Metrics

Each revised LS7 metric was coded as 2 if categorized as ideal, 1 if categorized as intermediate, and 0 if categorized as poor. Category 3 BMI-WHR (normal weight with central obesity) was defined as poor. The points for all revised LS7 metrics were then summed. Details of the cutoff values for each revised LS7 metric are described in Table 1. Participants with all available revised LS7 metrics were included in our study.

### Assessment of Outcomes

The primary outcome was all-cause mortality, and the secondary outcomes were cause-specific (cancer and CVD) mortality. Participants in the NHANES III (1988-1994) were prospectively followed from the date of enrollment until December 31, 2011.^15^ The National Center for Health Statistics ascertained mortality data from National Death Index death certificate records by matching the following data: Social Security number, name, date of birth, race/ethnicity, sex, state of birth, and state of residence.^16^ The cause of death was classified as cancer (*International Statistical Classification of Diseases and Related Health Problems, Tenth Revision* codes C00-C97) or CVD (codes I00-I09, I11, I13, I20-I51).

### Statistical Analysis

The baseline characteristics and revised LS7 metrics are reported as weighted prevalence values and confidence intervals. The person-years at risk were calculated from the baseline date to death, loss to follow-up, or December 31, 2011, whichever came first. Trends were tested using a logistic regression analysis that included a time variable equal to the median of the cycles after adjusting for age, sex, and race/ethnicity. Standard errors were estimated using Taylor series linearization.^17^

Participants in the NHANES III (1988-1994) were used for the association analysis. Cox proportional hazards models were used to estimate the hazard ratios (HRs) and 95% confidence intervals for single and combined revised LS7 metrics after adjusting for age, sex, and race/ethnicity. Additional adjustments included education (<12 or ≥12 years), alcohol intake (0, <3, or ≥3 drinks per week; 1 drink was defined as 12 oz of beer, 4 oz of wine, or 1 oz of hard liquor), smoking, physical activity, BMI, WHR, healthy diet score, total cholesterol, systolic BP, diastolic BP, and hemoglobin A_1c_ values. For additional adjustment, when we investigated the association between each revised LS7 metric and outcomes, the relevant variables were excluded. For example, when the association between goal levels of untreated BP and outcomes was analyzed, systolic BP and diastolic BP were not included in the model.

Kaplan-Meier curves for all-cause and cause-specific mortality were generated using the numbers of ideal revised LS7 metrics and compared using the log-rank test. Proportional hazards assumptions were evaluated by statistically testing the significance of time-dependent interaction terms. Derivations from proportionality were not observed.

The PAF describes the attributable risk of mortality during follow-up due to nonadherence to each revised LS7 metric and the revised combined metrics, with adjustment for age and sex.^18^ The sensitivity analyses were stratified by sex (male or female), age (<60 or ≥60 years), race/ethnicity (non-Hispanic white, non-Hispanic black, Mexican American, or other), education (<12 or ≥12 years), and alcohol drinking (0, <3, or ≥3 drinks per week). Interactions were performed using the likelihood ratio test with and without the cross-product interaction term. Another sensitivity analysis excluding death during the first 5 years of follow-up was conducted to assess whether the results had been influenced by reverse causation.

Sample weights were used for all analyses. Data were analyzed using SAS statistical software version 9.4 (SAS Institute). Statistical significance was set at *P* < .05 using a 2-sided test.

## Results

Data were available for 13 606 adults in NHANES III (1988-1994) (7329 [53%] female; mean [SD] age, 47 [17.7] years), 6360 in 1999 to 2004 (3442 [54%] female; mean [SD] age, 47 [18.6] years), 10 618 in 2005 to 2010 (5428 [51%] female; mean [SD] age, 47 [17.5] years), and 10 773 in 2011 to 2016 (5474 [50%] female; mean [SD] age, 48 [17.4] years). Participants in the NHANES III (1988-1994), who were younger, female, more educated, more likely to be non-Hispanic white, and less likely to consume alcohol than participants in other cycles, were more likely to have a greater number of ideal revised LS7 metrics (eTable 2 in the Supplement).

The characteristic distributions of the revised LS7 metrics for all survey cycles are shown in Table 2 and eTable 3 in the Supplement. Generally, less than 1% of the participants met all 6 to 7 ideal revised LS7 metrics, while approximately 21.8% to 30.7% met 2 or 3 of the ideal metrics (Table 2). From NHANES III (1988-1994) to 2011 to 2016, the weighted prevalence values of never smoking increased, whereas those of the ideal physical activity level, healthy diet score, and untreated fasting blood glucose level less than 100 mg/dL decreased (Table 2).

**Table 2.** Weighted Prevalence of Meeting Revised Life's Simple 7 Metrics in Adults, NHANES III (1988-1994), 1999-2004, 2005-2010, and 2011-2016

Life's Simple 7 Metrics^a^	NHANES III, 1988-1994		NHANES 1999-2004		NHANES 2005-2010		NHANES 2011-2016		*P* for Trend^b^
	Respondents, No.	Prevalence, % (95% CI)	Respondents, No.	Prevalence, % (95% CI)	Respondents, No.	Prevalence, % (95% CI)	Respondents, No.	Prevalence, % (95% CI)	
Smoking status									
Never	6731	46.0 (44.3-47.7)	3460	53.1 (50.2-56.0)	6023	56.4 (54.4-58.3)	6679	61.0 (59.4-62.5)	<.001
Former	3245	24.4 (23.1-25.7)	1680	25.0 (22.7-27.1)	2484	23.2 (21.9-24.4)	2287	23.0 (21.6-24.4)	<.001
Current	3630	29.4 (27.7-31.1)	1220	21.9 (19.9-23.7)	2111	20.4 (18.8-21.9)	1807	16.0 (14.6-17.2)	<.001
Physical activity^c^									
Ideal	4008	31.0 (29.0-33.1)	992	16.7 (14.9-18.3)	459	5.00 (4.27-5.76)	2749	27.6 (26.2-29.0)	<.001
Intermediate	6718	54.9 (53.2-56.5)	2506	47.0 (44.7-49.1)	1433	17.8 (15.3-20.1)	2842	29.8 (28.0-31.4)	<.001
Poor	2880	14.0 (12.4-15.5)	2862	36.3 (33.7-38.9)	8726	77.2 (74.4-79.9)	5182	42.6 (40.2-44.8)	<.001
BMI-WHR^d^									
1	1839	17.2 (15.8-18.6)	NA	NA	NA	NA	NA	NA	NA
2	2792	23.4 (22.2-24.7)	NA	NA	NA	NA	NA	NA	NA
3	639	3.89 (3.35-4.43)	NA	NA	NA	NA	NA	NA	NA
4	507	4.21 (3.59-4.83)	NA	NA	NA	NA	NA	NA	NA
5	2542	18.9 (18.0-19.9)	NA	NA	NA	NA	NA	NA	NA
6	1753	10.0 (9.27-10.8)	NA	NA	NA	NA	NA	NA	NA
7	151	1.09 (0.83-1.35)	NA	NA	NA	NA	NA	NA	NA
8	1501	9.83 (9.00-10.6)	NA	NA	NA	NA	NA	NA	NA
9	1882	11.1 (10.4-11.9)	NA	NA	NA	NA	NA	NA	NA
Healthy diet score^e^									
>69.3	4255	33.9 (32.2-35.6)	413	6.00 (4.89-6.97)	1248	11.9 (10.6-13.1)	1447	13.9 (12.8-15.1)	<.001
56.9-69.3	4521	32.9 (31.3-34.5)	1070	14.8 (13.2-16.4)	2459	22.7 (21.4-23.9)	2414	22.9 (21.7-24.2)	<.001
<56.9	4830	33.0 (31.0-35.0)	4877	79.2 (76.9-81.4)	6911	65.4 (63.2-67.5)	6912	63.0 (61.1-64.8)	<.001
Total serum cholesterol, mg/dL									
<200 (Untreated)^f^	6853	51.4 (49.4-53.4)	2905	47.2 (45.2-49.1)	5292	50.1 (48.6-51.4)	5822	53.3 (51.5-55.1)	<.001
200-239 or treated to goal	4115	30.0 (28.6-31.5)	2275	34.6 (33.0-36.1)	3574	33.4 (32.1-34.6)	3476	32.5 (31.0-33.9)	.68
≥240	2638	18.4 (17.1-19.7)	1180	18.2 (16.7-19.6)	1752	16.5 (15.5-17.5)	1475	14.2 (13.2-15.1)	<.001
Blood pressure, mm Hg									
<120/80 (Untreated)^f^	5470	46.3 (44.3-48.2)	2300	42.5 (40.1-44.7)	4569	46.7 (45.0-48.3)	4758	47.2 (45.4-49.0)	<.001
SBP 120-129 or DBP <80 or treated to goal	5120	33.7 (32.1-35.2)	2751	37.5 (35.7-39.3)	4306	37.3 (35.8-38.8)	4290	38.0 (36.2-39.6)	.13
SBP ≥130 or DBP ≥80	3016	19.9 (18.6-21.2)	1309	20.0 (18.4-21.6)	1743	16.0 (14.9-17.0)	1725	14.8 (13.5-16.1)	<.001
Fasting blood glucose, mg/dL^g^									
<100	3451	72.8 (70.3-75.4)	1970	67.4 (65.4-69.4)	2661	57.3 (54.6-60.0)	1892	57.6 (54.9-60.3)	<.001
100-125	1582	20.7 (18.8-22.7)	945	26.6 (24.6-28.5)	1951	36.2 (33.7-38.8)	1238	35.2 (32.7-37.6)	<.001
≥126	551	6.33 (5.12-7.54)	283	6.00 (4.82-7.06)	488	6.50 (5.58-7.32)	327	7.20 (5.96-8.41)	<.001
Ideal Life's Simple 7 metrics									
0	562	3.08 (2.71-3.46)	324	3.60 (2.94-4.25)	626	4.40 (3.88-4.89)	199	1.70 (1.39-1.99)	.07
1	1952	11.7 (10.6-12.7)	1266	16.9 (15.3-18.4)	2101	17.6 (16.3-18.7)	1322	11.8 (10.9-12.7)	.03
2	3216	21.8 (20.4-23.1)	1964	30.7 (28.9-32.5)	3084	28.7 (27.6-29.7)	2865	25.4 (24.0-26.7)	<.001
3	3389	25.0 (23.9-26.0)	1696	28.4 (26.6-30.1)	2895	29.4 (28.5-30.3)	3251	30.3 (28.9-31.6)	<.001
4	2643	21.5 (20.5-22.6)	930	17.0 (15.5-18.4)	1700	17.4 (16.0-18.7)	2318	22.6 (28.9-31.6)	.23
5	1349	12.0 (10.8-13.2)	176	3.30 (2.42-4.09)	208	2.50 (2.18-2.89)	732	7.20 (6.28-8.00)	<.001
6	427	3.93 (3.23-4.62)	4	0.10 (0-0.19)	4	0 (0-0)	86	1.00 (0.79-1.28)	<.001
7^h^	68	0.81 (0.53-1.09)	NA	NA	NA	NA	NA	NA	NA

Abbreviations: BMI-WHR, body mass index–waist hip ratio; DBP, diastolic blood pressure; NA, not available; NHANES, National Health and Nutrition Examination Survey; SBP, systolic blood pressure.

SI conversion factors: To convert total serum cholesterol to mmol/L, multiply by 0.0259; fasting blood glucose to mmol/L, mulitply by 0.0555.

^a^ All nonpregnant participants older than 20 years with available revised Life's Simple 7 metrics were included.

^b^ Trends across different surveys were analyzed by logistic regression model adjusted for age, sex, and race/ethnicity.

^c^ For the NHANES III (1988-1994), ideal physical activity (physically active) was defined using the following cutoffs: a metabolic equivalent score of 3 to 5.9 and participation 5 or more times per week, or a metabolic equivalent score of 6.0 or greater and participation 3 or more times per week. None was defined as poor. For the NHANES 1999 to 2014, 75 minutes or more of vigorous activity or 150 minutes or more of moderate activity or 150 minutes or more of combined moderate and vigorous physical activity; intermediate, more than 0 minutes of physical activity but less than recommendations; and poor, 0 minutes of physical activity. Physical activities included walking, jogging or running, bicycling, swimming, aerobics or aerobic dancing, other dancing, calisthenics, gardening or yard work, and other sports. The duration (minutes) of physical activity changed largely at NHANES 2007 to 2008; trend analysis was limited to NHANES 1999 to 2004 and NHANES 2005 to 2006.

^d^ A BMI-WHR level of 3 or greater (normal weight with central obesity) was defined as poor; all other levels were defined as ideal. The NHANES 1999 to 2016 did not measure hip circumference, and therefore the WHR is not available for the NHANES 1999 to 2004, 2005 to 2010, and 2011 to 2016. Different combinations of the WHR and BMI were only available for NHANES III (1988-1994).The classification of BMI combined with WHR was based on the work of Sahakyan et al.^2^

^e^ The healthy diet score was calculated based on the healthy eating index advocated by Dietary Guidelines for Americans^7^. The tertiles of the index were classified as ideal, intermediate, and poor, respectively.

^f^ Untreated value.

^g^ Fasting glucose level was available for a subgroup of participants in NHANES III (1988-1994) and NHANES 1999 to 2016. For trends, hemoglobin A_1c_ values less than 5.7% and 6.0% were used as proxies for fasting glucose levels less than 100 mg/dL and 125 mg/dL, respectively.

^h^ Values for WHR were not available in NHANES 1999-2016.

**Figure 1.**
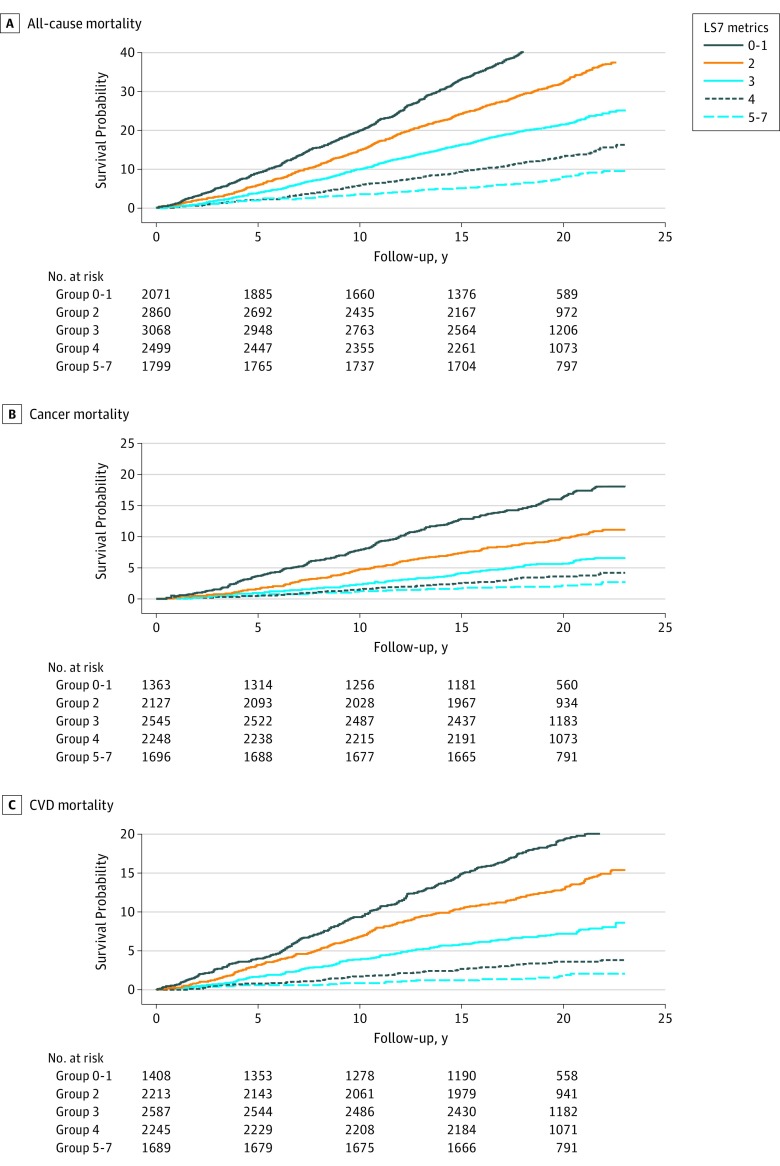
All-Cause and Cause-Specific Mortality by Revised Life's Simple 7 (LS7) Metrics Data are from the National Health and Nutrition Examination Survey III (1988-1994) linked mortality file.

A total of 12 299 participants were included in the association analysis in NHANES III (1988-1994). Of these participants, 4569 died during a median (range) follow-up of 19.16 (14.91-20.75) years (3015 all-cause deaths, 695 cancer deaths, and 859 CVD deaths). The Kaplan-Meier survival curves for participants who met 0 to 1, 2, 3, 4, or 5 to 7 ideal revised LS7 metrics are presented in Figure 1. Participants who met 5 to 7 ideal revised LS7 metrics had significantly lower cumulative incidence rates of all-cause and cause-specific mortality compared with other groups (*P* < .001 for all log-rank tests).

**Figure 2.**
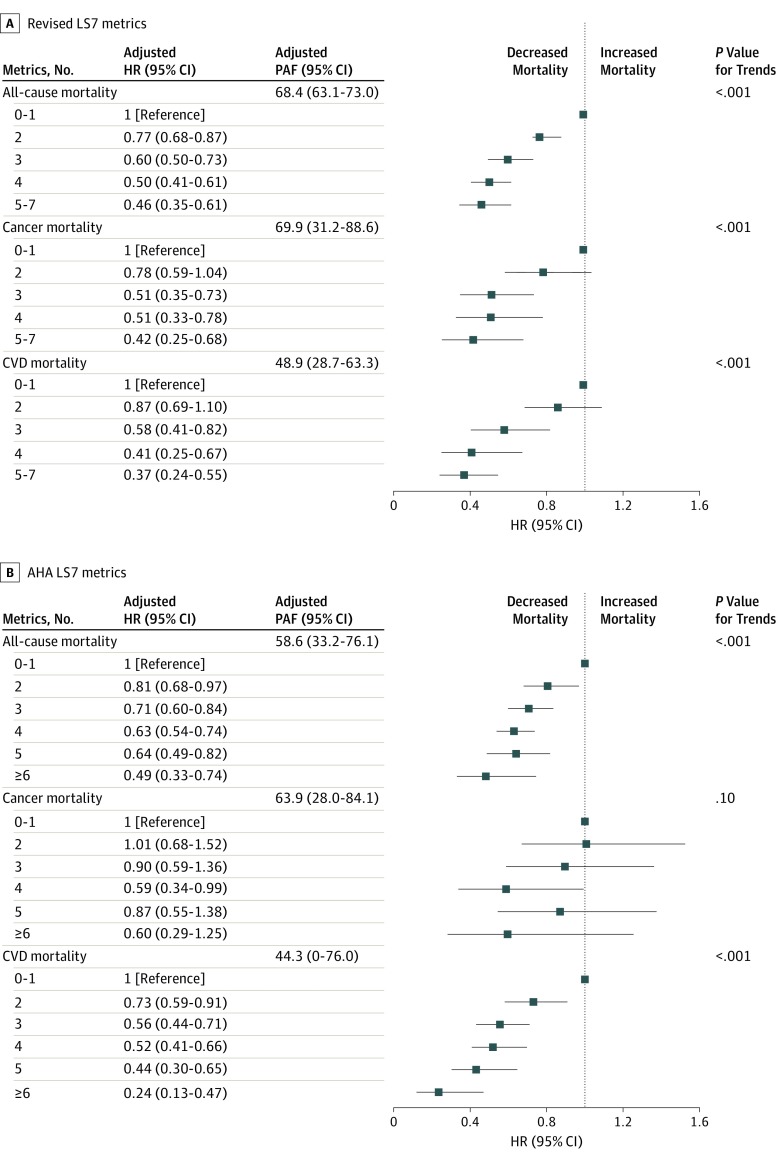
Adjusted Hazard Ratios (HRs) and Population-Attributable Fractions (PAFs) for All-Cause and Cause-Specific Mortality by Revised Life's Simple 7 (LS7) Metrics and American Heart Association (AHA) LS7 Metrics Because a small proportion of participants met none or all of the LS7 metrics, the revised metrics were collapsed into 5 groups (0-1, 2, 3, 4, and 5-7). The PAF for the revised LS7 metrics was adjusted for age and sex. The PAF for the AHA-recommended LS7 metrics was adjusted for age, sex, race/ethnicity, educational attainment, alcohol intake, family history of cardiovascular disease (CVD), smoking status, physical activity, body mass index, healthy diet score, total cholesterol level, blood pressure, and hemoglobin A_1c_ value.

Table 3 and Figure 2 and eFigure 1 and eFigure 2 in the Supplement present the adjusted HRs for all mortality outcomes according to the revised LS7 metrics and AHA-recommended LS7 metrics.^3^ Both metrics exhibited inverse dose-response associations with all-cause or cause-specific mortality (all *P* for trend < .001), except the association between the AHA-recommended LS7 metrics and cancer mortality (*P* for trend = .10) (Figure 2). Compared with participants who met 0 to 1 of the ideal revised LS7 metrics, the adjusted HRs of participants who met at least 5 metrics were 0.46 (95% CI, 0.35-0.61) for all-cause mortality, 0.42 (95% CI, 0.25-0.68) for cancer mortality, and 0.37 (95% CI, 0.24-0.55) for CVD mortality (Figure 2). The adjusted HRs of participants who met 6 or more AHA-recommended ideal LS7 metrics were 0.49 (95% CI, 0.33-0.74) for all-cause mortality, 0.60 (95% CI, 0.29-1.25) for cancer mortality, and 0.24 (95% CI, 0.13-0.47) for CVD mortality (Figure 2).

**Table 3.** Multivariable-Adjusted HRs and Population-Attributable Fractions for All-Cause and Cause-specific Mortality by Revised Life's Simple 7 Metrics^a^

Factor	All-Cause Mortality						Cancer Mortality						CVD Mortality					
	Cases/Total, No.	Adjusted HR (95% CI)^b^	*P* Value	Fully Adjusted HR (95% CI)^c^	*P* Value	PAF (95% CI)^d^	Cases/ Total, No.	Adjusted HR (95% CI)^b^	*P* Value	Fully Adjusted HR (95% CI)^c^	*P* Value	PAF (95% CI)^d^	Cases/Total, No.	Adjusted HR (95% CI)^b^	*P* Value	Fully Adjusted HR (95% CI)^c^	*P* Value	PAF (95% CI)^d^
Smoking status						15.2 (12.2-18.0)						28.83 (21.0-35.9)						10.7 (4.0-17.0)
Current	833/3377	1 [Reference]		1 [Reference]			242/2786	1 [Reference]		1 [Reference]			199/2743	1 [Reference]		1 [Reference]		
Former	933/2723	0.48 (0.41-0.56)	<.001	0.47 (0.39-0.57)	<.001		218/2008	0.39 (0.29-0.54)	<.001	0.41 (0.29-0.58)	<.001		275/2065	0.35 (0.26-0.47)	<.001	0.29 (0.21-0.42)	<.001	
Never	1249/6199	0.41 (0.35-0.48)	<.001	0.43 (0.37-0.50)	<.001		235/5185	0.25 (0.18-0.37)	<.001	0.25 (0.17-0.36)	<.001		385/5335	0.33 (0.24-0.46)	<.001	0.32 (0.22-0.47)	<.001	
Physical activity						4.26 (0.7-7.7)						NA						5.91 (0.03-14.1)
Poor	789/2437	1 [Reference]		1 [Reference]			163/1811	1 [Reference]		1 [Reference]			246/1894	1 [Reference]		1 [Reference]		
Intermediate	1300/6197	0.63 (0.54-0.73)	<.001	0.79 (0.66-0.94)	.01		304/5201	0.67 (0.48-0.95)	.03	1.04 (0.71-1.54)	.81		350/5247	0.55 (0.43-0.72)	<.001	0.74 (0.54-1.02)	.07	
Ideal	926/3665	0.59 (0.52-0.68)	<.001	0.82 (0.68-0.98)	.03		228/2967	0.68 (0.47-0.97)	.04	1.19 (0.76-1.86)	.43		263/3002	0.51 (0.39-0.65)	<.001	0.68 (0.47-0.99)	.049	
BMI-WHR^e^						16.61 (5.2-26.6)						NA						NA
3	307/526	1 [Reference]		1 [Reference]			54/273	1 [Reference]		1 [Reference]			108/327	1 [Reference]		1 [Reference]		
1	154/1790	0.56 (0.37-0.84)	.006	0.69 (0.42-1.13)	.14		40/1676	0.56 (0.29-1.09)	.09	0.87 (0.39-1.90)	.72		41/1677	0.52 (0.28-0.95)	.03	0.77 (0.33-1.79)	.54	
2	576/2530	0.70 (0.55-0.88)	.004	0.78 (0.58-1.05)	.11		151/2105	0.80 (0.51-1.26)	.34	1.10 (0.68-1.80)	.68		142/2096	0.55 (0.37-0.80)	.003	0.59 (0.37-0.94)	.03	
4	43/482	0.63 (0.41-0.98)	.04	1.00 (0.58-1.73)	.98		9/448	0.68 (0.26-1.76)	.42	1.40 (0.49-4.04)	.52		11/450	0.55 (0.23-1.28)	.16	1.19 (0.43-3.24)	.73	
5	385/2361	0.51 (0.39-0.66)	<.001	0.61 (0.44-0.85)	.004		87/2063	0.48 (0.28-0.84)	.01	0.70 (0.38-1.29)	.25		104/2080	0.37 (0.22-0.63)	.001	0.44 (0.23-0.83)	.01	
6	708/1472	0.81 (0.65-1.00)	.06	0.86 (0.68-1.10)	.24		166/930	0.87 (0.50-1.53)	.64	1.16 (0.63-2.13)	.62		200/964	0.67 (0.47-0.95)	.03	0.76 (0.52-1.10)	.15	
7	15/142	0.71 (0.34-1.47)	.35	0.88 (0.43-1.79)	.72		6/133	1.34 (0.35-5.09)	.65	1.96 (0.35-11.04)	.44		3/130	0.30 (0.07-1.15)	.08	0.32 (0.06-1.58)	.16	
8	259/1388	0.80 (0.63-1.02)	.07	0.91 (0.68-1.21)	.54		65/1194	0.99 (0.58-1.69)	.97	1.31 (0.78-2.22)	.29		69/1198	0.52 (0.32-0.84)	.01	0.50 (0.26-0.96)	.04	
9	568/1608	1.00 (0.80-1.25)	.95	0.96 (0.73-1.26)	.76		117/1157	1.00 (0.62-1.61)	.99	1.08 (0.63-1.85)	.76		181/1221	0.92 (0.61-1.39)	.71	0.86 (0.51-1.45)	.57	
Healthy diet score						8.0 (4.7-11.1)						11.7 (2.0-20.4)						NA
<56.9	1065/4426	1 [Reference]		1 [Reference]			258/3619	1 [Reference]		1 [Reference]			299/3660	1 [Reference]		1 [Reference]		
56.9-69.3	904/4119	0.81 (0.68-0.96)	.02	0.86 (0.73-1.01)	.08		213/3428	0.80 (0.56-1.13)	.21	0.83 (0.59-1.18)	.32		225/3440	0.80 (0.58-1.12)	.20	0.86 (0.60-1.24)	.43	
≥69.3	1046/3754	0.68 (0.59-0.78)	<.001	0.81 (0.70-0.93)	.005		224/2932	0.59 (0.43-0.81)	.002	0.72 (0.51-1.00)	.05		335/3043	0.69 (0.56-0.85)	<.001	0.91 (0.72-1.14)	.43	
Total serum cholesterol, mg/dL						NA						NA						10.1 (2.6-18.6)
≥240	848/2264	1 [Reference]		1 [Reference]			153/1569	1 [Reference]		1 [Reference]			290/1706	1 [Reference]		1 [Reference]		
200-239 or treated to goal	1017/3661	1.04 (0.91-1.19)	.51	1.08 (0.93-1.25)	.29		261/2905	1.68 (1.27-2.22)	<.001	1.72 (1.26-2.36)	.001		293/2937	0.90 (0.71-1.13)	.36	1.02 (0.81-1.28)	.86	
<200 (Untreated)	1150/6374	1.14 (0.99-1.31)	.07	1.15 (0.99-1.33)	.06		281/5505	1.66 (1.22-2.27)	.002	1.55 (1.09-2.21)	.02		276/5500	0.89 (0.66-1.21)	.48	0.91 (0.65-1.28)	.62	
Blood pressure, mm Hg						37.4 (32.8-42.5)						36.7 (26.8-28.9)						47.5 (38.2-57.3)
SBP ≥130 or DBP ≥80	938/2641	1 [Reference]		1 [Reference]			229/1932	1 [Reference]		1 [Reference]			289/1992	1 [Reference]		1 [Reference]		
SBP 120-129 or DBP <80 or treated to goal	1615/4429	0.96 (0.83-1.11)	.65	0.99 (0.85-1.16)	.95		336/3150	0.88 (0.61-1.29)	.53	0.82 (0.56-1.19)	.30		482/3296	0.88 (0.70-1.10)	.26	0.83 (0.64-1.08)	.16	
<120/80 (Untreated)	462/5229	0.66 (0.56-0.79)	<.001	0.73 (0.61-0.88)	.002		130/4897	0.67 (0.48-0.93)	.02	0.59 (0.41-0.84)	.004		88/4855	0.49 (0.36-0.65)	<.001	0.56 (0.38-0.81)	.003	
Fasting blood glucose, mg/dL^e^						6.8 (4.6-8.9)						2.8 (−3.2 to 8.6)						11.6 (6.1-16.8)
≥126	486/848	1 [Reference]		1 [Reference]			85/447	1 [Reference]		1 [Reference]			151/513	1 [Reference]		1 [Reference]		
100-125	868/2376	0.60 (0.49-0.73)	<.001	0.69 (0.54-0.87)	.003		207/1715	0.65 (0.45-0.95)	.03	0.81 (0.52-1.27)	.37		277/1785	0.49 (0.36-0.67)	<.001	0.65 (0.47-0.91)	.01	
<100	1661/9075	0.46 (0.39-0.55)	<.001	0.60 (0.47-0.76)	<.001		403/7817	0.53 (0.37-0.76)	.001	0.74 (0.45-1.21)	.23		431/7845	0.37 (0.26-0.51)	<.001	0.51 (0.35-0.75)	.001	

Abbreviations: BMI-WHR, body mass index–waist to hip ratio; DBP, diastolic blood pressure; HR, hazard ratio; NA, not available; NHANES, National Health and Nutrition Examination Survey; PAF, population-attributable fraction; SBP, systolic blood pressure.

SI conversion factors: To convert total serum cholesterol to mmol/L, multiply by 0.0259; fasting blood glucose to mmol/L, mulitply by 0.0555.

^a^ Data are from the NHANES III (1988-1994) linked mortality file. All nonpregnant participants older than 20 years with available revised Life's Simple 7 metrics were included.

^b^ Weighted prevalence and 95% CIs. Adjusted for age, sex, and race/ethnicity.

^c^ Weighted prevalence and 95% CIs. Adjusted for age, sex, race/ethnicity, educational attainment, alcohol intake, smoking status, physical activity, BMI, WHR, healthy diet score, total cholesterol level, SBP, DBP, and hemoglobin A_1c_ value as appropriate. For additional adjustment, when we investigate the association between each revised Life’s Simple 7 metric and outcomes, the relevant variables were excluded; for example, when the association between goal levels of untreated blood pressure and outcomes was analyzed, SBP and DBP were not included in the model.

^d^ Adjusted for age and sex. The individual PAFs cannot be calculated for the Life's Simple 7 metrics with adjusted hazard ratios of 1.0 or greater.

^e^ The classification of BMI-WHR was based on the work of Sahakyan et al.^2^

Of the individual revised LS7 metrics, never smoking and an untreated BP less than 120/80 mm Hg were associated with an independent lower risk for all outcomes. A BMI-WHR level of 5 (BMI ≤29.9 without central obesity) and untreated fasting blood glucose level less than 100 mg/dL were associated with an independent lower risk for all-cause and CVD mortality. A healthy diet score 69.3 or higher was associated with an independent lower risk for all-cause mortality. By contrast, an untreated total cholesterol less than 200 mg/dL was associated with an independent higher risk for cancer mortality (Table 3).

We also performed stratified analyses by age, sex, race/ethnicity, education, and alcohol consumption (eTables 4, 5, 6, 7, and 8 in the Supplement). Significant interaction between the revised LS7 metrics and age was observed for cancer mortality (eFigure 3 in the Supplement; *P* for interaction = .002), and significant interactions between the revised LS7 metrics and education were observed for all-cause and CVD mortality (*P* for interaction = .03 and *P* for interaction = .04, respectively). The associations between the number of revised LS7 metrics and all outcomes were more pronounced in younger participants (eTable 4 in the Supplement) and female participants (eTable 5 in the Supplement). In another sensitivity test, 551 participants whose outcomes occurred during the first 5 years of follow-up were excluded. The results in the remaining sample remained similar to those observed in the full sample (data not shown).

Table 3 also presents the PAF for the individual revised ideal LS7 metrics. The PAF analysis indicated that 68.4% (95% CI, 63.1%-73.0%) of all-cause mortality, 69.9% (95% CI, 31.2%-88.6%) of cancer mortality, and 48.9% (95% CI, 28.7%-63.3%) of CVD mortality were associated with nonadherence to the revised combined ideal LS7 metrics (Figure 2), whereas 58.6% (95% CI, 33.2%-76.1%) of all-cause mortality, 63.9% (95% CI, 28.0%-84.1%) of cancer mortality, and 44.3% (95% CI, 0%-76.0%) of CVD mortality were associated with nonadherence to the combined AHA-recommended ideal LS7 metrics (Figure 2). Blood pressure was the most significant individual factor associated with all-cause mortality (PAF = 37.4%), cancer mortality (PAF = 36.7%), and CVD mortality (PAF = 47.5%) (Table 3; eFigure 2 in the Supplement).

## Discussion

Compared with the AHA-derived LS7, the revised LS7 more clearly demonstrated an associated reduction in the risk of cancer mortality. No significant dose-response association was observed between the AHA-derived LS7 metrics and cancer mortality, suggesting that the revised metrics are more strongly associated with cancer mortality than the AHA-derived metrics. Abdominal fat, such as visceral adipose tissue and subcutaneous adipose tissue, was positively associated with all-cause and cancer mortality rather than the CVD-related death in a prospective study.^19^ The diet components recommended by AHA only focused on the promotion of cardiovascular health^1^; however, dietary components in the Healthy Eating Index–2010 are more comprehensive.^13^ This may partly explain why the combined revised LS7 metrics are more strongly associated with cancer mortality.

A 68.4%, 69.9%, and 48.9% reduction in relative risk of all-cause, cancer, and CVD mortality could be attributed to the ideal revised LS7 metrics, respectively. In the fully adjusted model, participants with a BMI less than or equal to 29.9 but without central obesity had a decreased risk of all-cause and CVD mortality. However, the AHA recommendation of a BMI less than 25 was not associated with all-cause and CVD mortality in a previous study.^3^ Using the same data, central obesity was found to correlate with a higher risk of CVD mortality in normal-weight participants compared with those without central obesity (in BMI category).^14^ Similarly, a higher risk of mortality was observed in patients with coronary artery disease and a large WHR regardless of BMI.^20^ In our study, we observed only a weak correlation of BMI with WHR (correlation coefficient = 0.21), implying that WHR and BMI provide different information.

Our findings further indicate the additional significance of the WHR beyond BMI. The WHR is a robust indicator of visceral fat, whereas the BMI reflects general adiposity. An increased WHR parallels either a greater accumulation of the intra-abdominal fat mass or a greater decrease in the gluteofemoral muscle mass,^21^ and intra-abdominal fat has been associated with CVD mortality.^21^ Overweight or obese participants may carry a greater proportion of subcutaneous fat in the hips and legs, which is less associated with adverse outcomes compared with visceral fat.^22^ In contrast to subcutaneous fat, visceral adipose tissue is associated more strongly with adverse metabolic risk factors independent of BMI and waist circumference.^23^

A healthy diet score greater than or equal to 69.3 was independently associated with a reduced risk of all-cause mortality in our study. However, the achievement of 2 or more dietary components as defined by the AHA was not associated with all-cause mortality.^12^ Neither a healthy diet score of 69.3 or greater or 2 or more dietary components was found to be associated with CVD and cancer mortality in a previous study.^12^ Most studies of the healthy diet score considered the separate components of diets. However, as dietary components act collaboratively rather than individually, the dietary pattern is more representative of the total quality than the individual intakes.

A total cholesterol level less than 200 mg/dL was independently associated with a higher risk of cancer mortality in our study. Consistent with our findings, other researchers reported an inverse association between a low serum cholesterol level and increased cancer mortality.^24,25^ However, unknown and unmeasured confounding factors affecting mortality and preexisting disease at baseline might contribute to the observed increase in mortality.^26^ Still, the inverse association in our study remained after further excluding participants whose outcomes occurred during the first 5 years of follow-up.

In our study, BP was the most significant personal contributor, associated with 36.7% or more of the PAF. An untreated BP less than 120/80 mm Hg was associated with a reduced risk of all-cause and CVD mortality under both the AHA-defined and revised LS7 metrics.^3^ Untreated BP less than 120/80 mm Hg was associated with an HR for all-cause mortality of 0.73 (95% CI, 0.61-0.88) and HR for CVD mortality of 0.56 (95% CI, 0.38-0.81) in our study, in contrast with 0.81 (95% CI 0.68–0.95) for all-cause mortality and 0.64 (95% CI, 0.47-0.86) for CVD mortality in the study by Yang et al.^3^

Participants who were younger and female were more likely to meet a greater number of ideal LS7 health metrics. The significant interactions observed between the ideal revised LS7 metrics and age on cancer mortality, and between the ideal revised LS7 metrics and education on all-cause and CVD mortality, suggest that the LS7 should be promoted more strongly among high-risk groups.

When reestablishing our revised LS7 metrics, we emphasized the conjunction of WHR and BMI, an integrated dietary pattern (Healthy Eating Index–2010), and a new BP threshold for hypertension.^8^ A strength of our study involves the use of standardized data from a large representative sample of US adults. The NHANES applies stronger criteria to guarantee minimal nonsampling and measurement errors during survey planning, data collection, and processing. A sensitivity analysis ensured the reliability of our findings. We carefully adjusted for potential confounding factors and applied stringent exclusion criteria to reduce potential bias due to reverse causation.

### Limitations

This study has some limitations, so our findings should be interpreted cautiously. The comparison is not direct, because few participants met all of the 6 to 7 revised LS7 metrics in our study, so 7 groups were collapsed into 5 groups (0-1, 2, 3, 4, and 5-7); however, 6 groups (0-1, 2, 3, 4, 5, and 6-7) were classified for the AHA-recommended version.^3^ Second, the physical activity levels, smoking history, and dietary records were self-reported in the NHANES surveys, which may have introduced recall bias. Third, the revised LS7 metrics were only available at baseline, and long-term changes (eg, trajectories) in LS7 metrics could not be captured. Misclassification error of underlying and contributing causes of death and residual confounding and competing risks for cause-specific mortalities should also be noted. In addition, because genetic data were not available, the potential impact of genetic backgrounds in participants of different races/ethnicities on the association between revised LS7 metrics and risks of mortality outcomes were not investigated. Furthermore, the potential misclassification of the scoring approach in our study could not be avoided.

## Conclusions

Our study indicated that few US adults met 6 to 7 ideal revised LS7 metrics. For participants with BMI less than or equal to 29.9 but without central obesity, the revised metrics were independently associated with decreased risk of all-cause and cardiovascular disease mortality. The individual revised LS7 metrics with modified criteria regarding weight, BP, and diet provide more information about factors associated with cancer mortality than the original AHA-derived LS7 metrics.

## Notes

Supplement. **eTable 1.** The Sex-Stratified Composite Classification of BMI and WHR **eTable 2.** Baseline Characteristics of Adults According to the Numbers of Revised Ideal Life's Simple 7 Metrics, NHANES III (1988-1994) **eTable 3.** Characteristics of US Adults—NHANES III (1988-1994), 1999-2004, 2005-2010, 2011-2016 **eTable 4.** Adjusted HRs (95% CIs) for All-Cause and Cause-Specific Mortality by Numbers of Revised Ideal Life's Simple 7 Metrics and Age Groups, NHANES III (1988-1994) Linked Mortality File-2011 **eTable 5.** Adjusted HRs (95% CIs) for All-Cause and Cause-Specific Mortality by Numbers of Revised Ideal Life's Simple 7 Metrics and Sex, NHANES III (1988-1994) Linked Mortality File-2011 **eTable 6.** Adjusted HRs (95% CIs) for All-Cause and Cause-Specific Mortality by Numbers of Revised Ideal Life's Simple 7 Metrics and Race/Ethnicity, NHANES III (1988-1994) Linked Mortality File-2011 **eTable 7.** Adjusted HRs (95% CIs) for All-Cause and Cause-Specific Mortality by Numbers of Revised Ideal Life's Simple 7 Metrics and Educational Attainment, NHANES III (1988-1994) Linked Mortality File-2011 **eTable 8.** Adjusted HRs (95% CIs) for All-Cause and Cause-Specific Mortality by Numbers of Revised Ideal Life's Simple 7 Metrics and Alcohol Groups, NHANES III (1988-1994) Linked Mortality File-2011 **eFigure 1.** Adjusted Hazard Ratios and Population Attributable Fractions for All-Cause and Cause-Specific Mortality by Revised Life's Simple 7 Metrics **eFigure 2.** The Adjusted Hazard Ratios and Population Attributable Fractions for All-Cause and Cause-Specific Mortality by Each Revised Life's Simple 7 Metrics and That Recommended by AHA **eFigure 3.** The Interaction Between Age, Education and LS7 Health Metrics on Mortality Outcomes

## References

[1] Lloyd-Jones DM, Hong Y, Labarthe D, ; American Heart Association Strategic Planning Task Force and Statistics Committee Defining and setting national goals for cardiovascular health promotion and disease reduction: the American Heart Association’s strategic Impact Goal through 2020 and beyond. Circulation. 2010;121(4):-. doi:10.1161/CIRCULATIONAHA.109.192703 20089546

[2] Sahakyan KR, Somers VK, Rodriguez-Escudero JP, Normal-weight central obesity: implications for total and cardiovascular mortality. Ann Intern Med. 2015;163(11):827-835. doi:10.7326/M14-2525 26551006 PMC4995595

[3] Yang Q, Cogswell ME, Flanders WD, Trends in cardiovascular health metrics and associations with all-cause and CVD mortality among US adults. JAMA. 2012;307(12):1273-1283. doi:10.1001/jama.2012.339 22427615 PMC9004324

[4] Younus A, Aneni EC, Spatz ES, A systematic review of the prevalence and outcomes of ideal cardiovascular health in US and non-US populations. Mayo Clin Proc. 2016;91(5):649-670. doi:10.1016/j.mayocp.2016.01.019 27040086

[5] Lin TY, Lim PS, Hung SC Impact of misclassification of obesity by body mass index on mortality in patients with CKD. Kidney Int Rep. 2017;3(2):447-455. doi:10.1016/j.ekir.2017.12.009 29725649 PMC5932305

[6] Flegal KM, Kit BK, Graubard BI Bias in hazard ratios arising from misclassification according to self-reported weight and height in observational studies of body mass index and mortality. Am J Epidemiol. 2018;187(1):125-134. doi:10.1093/aje/kwx193 29309516 PMC5859975

[7] Van Horn L, Carson JA, Appel LJ, ; American Heart Association Nutrition Committee of the Council on Lifestyle and Cardiometabolic Health; Council on Cardiovascular Disease in the Young; Council on Cardiovascular and Stroke Nursing; Council on Clinical Cardiology; and Stroke Council Recommended dietary pattern to achieve adherence to the American Heart Association/American College of Cardiology (AHA/ACC) guidelines: a scientific statement from the American Heart Association. Circulation. 2016;134(22):e505-e529. doi:10.1161/CIR.0000000000000462 27789558

[8] Whelton PK, Carey RM, Aronow WS, 2017 ACC/AHA/AAPA/ABC/ACPM/AGS/APhA/ASH/ASPC/NMA/PCNA Guideline for the Prevention, Detection, Evaluation, and Management of High Blood Pressure in Adults: executive summary: a report of the American College of Cardiology/American Heart Association Task Force on Clinical Practice Guidelines [published correction appears in *Hypertension*. 2018;71(6):e136-e139]. J Am Coll Cardiol. 2018;71(19):2199-2269. doi:10.1016/j.jacc.2017.11.005 29146533

[9] Wang ZJ, Zhou YJ, Galper BZ, Gao F, Yeh RW, Mauri L Association of body mass index with mortality and cardiovascular events for patients with coronary artery disease: a systematic review and meta-analysis. Heart. 2015;101(20):1631-1638. doi:10.1136/heartjnl-2014-307119 26025084

[10] Barton V, Armeson K, Hampras S, Nonmelanoma skin cancer and risk of all-cause and cancer-related mortality: a systematic review. Arch Dermatol Res. 2017;309(4):243-251. doi:10.1007/s00403-017-1724-5 28285366 PMC5396844

[11] Ainsworth BE, Haskell WL, Herrmann SD, 2011 Compendium of Physical Activities: a second update of codes and MET values. Med Sci Sports Exerc. 2011;43(8):1575-1581. doi:10.1249/MSS.0b013e31821ece12 21681120

[12] Beddhu S, Baird BC, Zitterkoph J, Neilson J, Greene T Physical activity and mortality in chronic kidney disease (NHANES III). Clin J Am Soc Nephrol. 2009;4(12):1901-1906. doi:10.2215/CJN.01970309 19820134 PMC2798872

[13] Krebs-Smith SM, Pannucci TE, Subar AF, Update of the Healthy Eating Index: HEI-2015. J Acad Nutr Diet. 2018;118(9):1591-1602. doi:10.1016/j.jand.2018.05.021 30146071 PMC6719291

[14] Sahakyan KR, Somers VK, Rodriguez-Escudero JP, Normal-weight central obesity: implications for total and cardiovascular mortality. Ann Intern Med. 2015;163(11):827-835. doi:10.7326/M14-2525 26551006 PMC4995595

[15] National Center for Health Statistics Office of Analysis and Epidemiology NCHS data linked to NDI mortality files. https://www.cdc.gov/nchs/data-linkage/mortality.htm. Accessed February 27, 2015.

[16] National Center for Health Statistics Office of Analysis and Epidemiology National Death Index. https://www.cdc.gov/nchs/ndi/index.htm. Accessed October 31, 2018.

[17] National Center for Health Statistics Analytic and reporting guidelines: National Health and Nutrition Examination Survey, NHANES III (1988-1994). https://wwwn.cdc.gov/nchs/nhanes/analyticguidelines.aspx. Published August 19, 2013. Accessed October 31, 2018.

[18] von Cube M, Schumacher M, Putter H, Timsit JF, van de Velde C, Wolkewitz M The population-attributable fraction for time-dependent exposures using dynamic prediction and landmarking [published online June 19, 2019]. Biom J. doi:10.1002/bimj.201800252 31216103

[19] Rosenquist KJ, Massaro JM, Pedley A, Fat quality and incident cardiovascular disease, all-cause mortality, and cancer mortality. J Clin Endocrinol Metab. 2015;100(1):227-234. doi:10.1210/jc.2013-4296 25226289 PMC5399496

[20] Coutinho T, Goel K, Corrêa de Sá D, Central obesity and survival in subjects with coronary artery disease: a systematic review of the literature and collaborative analysis with individual subject data. J Am Coll Cardiol. 2011;57(19):1877-1886. doi:10.1016/j.jacc.2010.11.058 21545944

[21] Larsson B, Svärdsudd K, Welin L, Wilhelmsen L, Björntorp P, Tibblin G Abdominal adipose tissue distribution, obesity, and risk of cardiovascular disease and death: 13 year follow up of participants in the study of men born in 1913. Br Med J (Clin Res Ed). 1984;288(6428):1401-1404. doi:10.1136/bmj.288.6428.1401 6426576 PMC1441047

[22] Manolopoulos KN, Karpe F, Frayn KN Gluteofemoral body fat as a determinant of metabolic health. Int J Obes (Lond). 2010;34(6):949-959. doi:10.1038/ijo.2009.286 20065965

[23] Fox CS, Massaro JM, Hoffmann U, Abdominal visceral and subcutaneous adipose tissue compartments: association with metabolic risk factors in the Framingham Heart Study. Circulation. 2007;116(1):39-48. doi:10.1161/CIRCULATIONAHA.106.675355 17576866

[24] Törnberg SA, Holm LE, Carstensen JM, Eklund GA Cancer incidence and cancer mortality in relation to serum cholesterol. J Natl Cancer Inst. 1989;81(24):1917-1921. doi:10.1093/jnci/81.24.1917 2593170

[25] Schuit AJ, Van Dijk CE, Dekker JM, Schouten EG, Kok FJ Inverse association between serum total cholesterol and cancer mortality in Dutch civil servants. Am J Epidemiol. 1993;137(9):966-976. doi:10.1093/oxfordjournals.aje.a116769 8317454

[26] Iribarren C, Reed DM, Burchfiel CM, Dwyer JH Serum total cholesterol and mortality: confounding factors and risk modification in Japanese-American men. JAMA. 1995;273(24):1926-1932. doi:10.1001/jama.1995.03520480046038 7783302

