# Supplementary material for: National Trends in American Heart Association Revised Life's Simple 7 Metrics Associated With Risk of Mortality Among US Adults
Source: JAMA Netw Open. 2019 Oct 11;2(10):e1913131. doi: 10.1001/jamanetworkopen.2019.13131 (PMC6804021; doi:10.1001/jamanetworkopen.2019.13131)
Supplement: Supplement. — eTable 1. The Sex-Stratified Composite Classification of BMI and WHR eTable 2. Baseline Characteristics of Adults According to the Numbers of Revised Ideal Life's Simple 7 Metrics, NHANES III (1988-1994) eTable 3. Characteristics of US Adults—NHANES III (1988-1994), 1999-2004, 2005-2010, 2011-2016 eTable 4. Adjusted HRs (95% CIs) for All-Cause and Cause-Specific Mortality by Numbers of Revised Ideal Life's Simple 7 Metrics and Age Groups, NHANES III (1988-1994) Linked Mortality File-2011 eTable 5. Adjusted HRs (95% CIs) for All-Cause and Cause-Specific Mortality by Numbers of Revised Ideal Life's Simple 7 Metrics and Sex, NHANES III (1988-1994) Linked Mortality File-2011 eTable 6. Adjusted HRs (95% CIs) for All-Cause and Cause-Specific Mortality by Numbers of Revised Ideal Life's Simple 7 Metrics and Race/Ethnicity, NHANES III (1988-1994) Linked Mortality File-2011 eTable 7. Adjusted HRs (95% CIs) for All-Cause and Cause-Specific Mortality by Numbers of Revised Ideal Life's Simple 7 Metrics and Educational Attainment, NHANES III (1988-1994) Linked Mortality File-2011 eTable 8. Adjusted HRs (95% CIs) for All-Cause and Cause-Specific Mortality by Numbers of Revised Ideal Life's Simple 7 Metrics and Alcohol Groups, NHANES III (1988-1994) Linked Mortality File-2011 eFigure 1. Adjusted Hazard Ratios and Population Attributable Fractions for All-Cause and Cause-Specific Mortality by Revised Life's Simple 7 Metrics eFigure 2. The Adjusted Hazard Ratios and Population Attributable Fractions for All-Cause and Cause-Specific Mortality by Each Revised Life's Simple 7 Metrics and That Recommended by AHA eFigure 3. The Interaction Between Age, Education and LS7 Health Metrics on Mortality Outcomes [file jamanetwopen-2-e1913131-s001.pdf]

Supplementary Online Content

Han L, You D, Ma W, et al. National trends in American Heart Association revised Life's Simple 7 metrics associated with risk of mortality among US adults. *JAMA Netw Open*. 2019;2(10):e1913131. doi:10.1001/jamanetworkopen.2019.13131

eTable 1. The Sex-Stratified Composite Classification of BMI and WHR

eTable 2. Baseline Characteristics of Adults According to the Numbers of Revised Ideal Life's Simple 7 Metrics, NHANES III (1988-1994)

eTable 3. Characteristics of US Adults—NHANES III (1988-1994), 1999-2004, 2005-2010, 2011-2016

eTable 4. Adjusted HRs (95% CIs) for All-Cause and Cause-Specific Mortality by Numbers of Revised Ideal Life's Simple 7 Metrics and Age Groups, NHANES III (1988-1994) Linked Mortality File-2011

eTable 5. Adjusted HRs (95% CIs) for All-Cause and Cause-Specific Mortality by Numbers of Revised Ideal Life's Simple 7 Metrics and Sex, NHANES III (1988-1994) Linked Mortality File-2011

eTable 6. Adjusted HRs (95% CIs) for All-Cause and Cause-Specific Mortality by Numbers of Revised Ideal Life's Simple 7 Metrics and Race/Ethnicity, NHANES III (1988-1994) Linked Mortality File-2011

eTable 7. Adjusted HRs (95% CIs) for All-Cause and Cause-Specific Mortality by Numbers of Revised Ideal Life's Simple 7 Metrics and Educational Attainment, NHANES III (1988-1994) Linked Mortality File-2011

eTable 8. Adjusted HRs (95% CIs) for All-Cause and Cause-Specific Mortality by Numbers of Revised Ideal Life's Simple 7 Metrics and Alcohol Groups, NHANES III (1988-1994) Linked Mortality File-2011

eFigure 1. Adjusted Hazard Ratios and Population Attributable Fractions for All-Cause and Cause-Specific Mortality by Revised Life's Simple 7 Metrics

eFigure 2. The Adjusted Hazard Ratios and Population Attributable Fractions for All-Cause and Cause-Specific Mortality by Each Revised Life's Simple 7 Metrics and That Recommended by AHA

eFigure 3. The Interaction Between Age, Education and LS7 Health Metrics on Mortality Outcomes

This supplementary material has been provided by the authors to give readers additional information about their work.

© 2019 Han L et al. *JAMA Network Open.*

eTable 1: The sex-stratified composite classification of BMI and WHR

BMI-WHR [a]	Male		Female	
	BMI	WHR	BMI	WHR
1	≤24.9	<0.9	≤24.9	<0.8
2	≤24.9	<1	≤24.9	<0.92
3	≤24.9	>1	≤24.9	>0.92
4	≤29.9	<0.9	≤29.9	<0.8
5	≤29.9	<1	≤29.9	<0.92
6	≤29.9	>1	≤29.9	>0.92
7	≥30	<0.9	≥30	<0.8
8	≥30	<1	≥30	<0.92
9	≥30	>1	≥30	>0.92

Abbreviations: BMI-WHR, body mass index-waist-hip ratio; BMI: Body Mass Index; WHR: Waist hip ratio

The classification of BMI combined with WHR was based on the reference 2 in the paper. BMI-WHR category 3 (normal weight with central obesity) was defined as “poor”.

© 2019 Han L et al. *JAMA Network Open.*

eTable 2: Baseline characteristics of adults according to the Numbers of revised ideal Life's Simple 7 Metrics, NHANES III (1988-1994)

Characteristics*	No. of participants	Numbers of revised ideal Life's Simple 7 metrics †								p-value‡
		0	1	2	3	4	5	6	7	
No. of participants	13606	562	1952	3216	3389	2643	1349	427	68	
**Age**										
<60	9722	0.63 (0.57-0.69)	0.65 (0.62-0.69)	0.73 (0.70-0.76)	0.79 (0.76-0.81)	0.88 (0.87-0.90)	0.93 (0.91-0.95)	0.95 (0.93-0.97)	0.99 (0.98-1.00)	<0.001
≥60	3884	0.36 (0.30-0.42)	0.34 (0.30-0.37)	0.26 (0.23-0.29)	0.20 (0.18-0.23)	0.11 (0.09-0.12)	0.06 (0.04-0.08)	0.04 (0.02-0.06)	0.00 (0.00-0.01)	<0.001
**Gender**										<0.001
Male	6539	0.66 (0.61-0.71)	0.59 (0.56-0.63)	0.53 (0.50-0.56)	0.49 (0.46-0.52)	0.45 (0.42-0.48)	0.37 (0.34-0.40)	0.36 (0.30-0.42)	0.36 (0.21-0.52)	
Female	7067	0.33 (0.28-0.38)	0.40 (0.36-0.43)	0.46 (0.43-0.49)	0.50 (0.47-0.53)	0.54 (0.51-0.57)	0.62 (0.59-0.65)	0.63 (0.57-0.69)	0.63 (0.47-0.78)	
**Race/Ethnicity**										<0.001
Non-Hispanic White	5263	0.72 (0.67-0.78)	0.75 (0.72-0.78)	0.76 (0.73-0.80)	0.75 (0.72-0.78)	0.73 (0.71-0.76)	0.74 (0.68-0.79)	0.77 (0.72-0.83)	0.83 (0.78-0.88)	
Non-Hispanic Black	3910	0.18 (0.15-0.21)	0.13 (0.11-0.15)	0.11 (0.10-0.13)	0.11 (0.09-0.12)	0.10 (0.08-0.11)	0.09 (0.07-0.11)	0.09 (0.06-0.12)	0.04 (0.02-0.06)	
Mexican American	3862	0.04 (0.03-0.05)	0.05 (0.04-0.06)	0.04 (0.04-0.05)	0.05 (0.04-0.06)	0.05 (0.04-0.07)	0.05 (0.04-0.07)	0.05 (0.02-0.07)	0.04 (0.01-0.06)	
Other	571	0.04 (0.00-0.09)	0.06 (0.04-0.08)	0.06 (0.04-0.08)	0.08 (0.05-0.10)	0.10 (0.07-0.12)	0.10 (0.06-0.15)	0.07 (0.03-0.10)	0.07 (0.05-0.09)	
**Years of education**										<0.001
<12	5425	0.43 (0.36-0.51)	0.37 (0.33-0.41)	0.28 (0.25-0.32)	0.25 (0.22-0.27)	0.18 (0.15-0.20)	0.14 (0.10-0.17)	0.08 (0.04-0.11)	0.07 (0.02-0.12)	

© 2019 Han L et al. *JAMA Network Open.*

12	6427	0.49 (0.40-0.57)	0.51 (0.47-0.54)	0.55 (0.53-0.57)	0.54 (0.51-0.57)	0.56 (0.53-0.60)	0.54 (0.50-0.58)	0.56 (0.51-0.61)	0.36 (0.26-0.46)	
≥12	1754	0.06 (0.03-0.10)	0.11 (0.08-0.14)	0.15 (0.12-0.18)	0.20 (0.17-0.23)	0.24 (0.21-0.28)	0.31 (0.26-0.36)	0.35 (0.29-0.41)	0.56 (0.47-0.64)	
**Alcohol intake (drinks/wk)**										0.007
None	6873	0.58 (0.51-0.66)	0.54 (0.49-0.59)	0.52 (0.49-0.55)	0.48 (0.44-0.51)	0.47 (0.43-0.51)	0.46 (0.42-0.51)	0.43 (0.36-0.51)	0.50 (0.33-0.66)	
<3/per week	2384	0.20 (0.13-0.27)	0.20 (0.16-0.23)	0.20 (0.17-0.23)	0.23 (0.20-0.26)	0.24 (0.21-0.28)	0.24 (0.20-0.28)	0.31 (0.24-0.39)	0.35 (0.20-0.50)	
⩾3/per week	2623	0.20 (0.16-0.25)	0.25 (0.20-0.29)	0.26 (0.23-0.30)	0.28 (0.25-0.31)	0.27 (0.23-0.31)	0.28 (0.24-0.32)	0.24 (0.18-0.30)	0.14 (0.03-0.25)	
**Life's Simple 7 Metrics**										
**Smoking status**										<0.001
Never	6731	NA	0.12 (0.10-0.14)	0.31 (0.28-0.33)	0.45 (0.42-0.48)	0.58 (0.55-0.61)	0.75 (0.71-0.79)	0.90 (0.86-0.94)	1.00 (1.00-1.00)	
Former	3245	0.46 (0.38-0.53)	0.44 (0.40-0.48)	0.32 (0.29-0.35)	0.23 (0.20-0.25)	0.17 (0.15-0.20)	0.08 (0.06-0.11)	0.04 (0.01-0.06)	NA	
Current	3630	0.53 (0.46-0.61)	0.42 (0.38-0.47)	0.36 (0.32-0.39)	0.31 (0.28-0.33)	0.23 (0.20-0.26)	0.15 (0.11-0.19)	0.05 (0.03-0.07)	NA	
**Physical activity**[§]										<0.001
revised ideal	4008	-	0.09 (0.07-0.11)	0.17 (0.15-0.19)	0.31 (0.28-0.34)	0.38 (0.35-0.41)	0.53 (0.49-0.58)	0.68 (0.59-0.76)	1.00 (1.00-1.00)	
Intermediate	6718	0.70 (0.63-0.77)	0.67 (0.63-0.70)	0.64 (0.62-0.67)	0.54 (0.52-0.57)	0.51 (0.48-0.54)	0.39 (0.35-0.42)	0.29 (0.20-0.37)	-	

© 2019 Han L et al. *JAMA Network Open.*

Poor	2880	0.29 (0.22-0.36)	0.23 (0.20-0.26)	0.17 (0.15-0.20)	0.13 (0.11-0.15)	0.09 (0.07-0.11)	0.07 (0.04-0.09)	0.02 (0.01-0.04)	-	
**BMI-WHR**[ǁ]										<0.001
1	1790	-	0.00 (0.00-0.01)	0.02 (0.01-0.03)	0.07 (0.06-0.08)	0.23 (0.20-0.26)	0.48 (0.43-0.53)	0.74 (0.67-0.81)	1.00 (1.00-1.00)	
2	2530	0.13 (0.09-0.16)	0.18 (0.15-0.21)	0.20 (0.17-0.22)	0.28 (0.26-0.30)	0.27 (0.25-0.30)	0.24 (0.20-0.28)	0.12 (0.08-0.17)	-	
3	526	0.07 (0.04-0.09)	0.05 (0.04-0.07)	0.05 (0.03-0.06)	0.03 (0.03-0.04)	0.02 (0.01-0.03)	0.02 (0.00-0.03)	0.00 (0.00-0.01)	-	
4	482	0.00 (0.00-0.01)	0.01 (0.00-0.02)	0.02 (0.01-0.03)	0.05 (0.04-0.06)	0.06 (0.04-0.07)	0.04 (0.03-0.06)	0.02 (0.00-0.04)	-	
5	2361	0.16 (0.10-0.22)	0.20 (0.16-0.23)	0.23 (0.21-0.25)	0.20 (0.18-0.22)	0.18 (0.16-0.21)	0.11 (0.08-0.14)	0.06 (0.03-0.09)	-	
6	1472	0.21 (0.15-0.26)	0.17 (0.14-0.19)	0.16 (0.14-0.18)	0.09 （0.08-0.11）	0.05 (0.03-0.06)	0.02 (0.01-0.03)	0.00 (0.00-0.01)	-	
7	142	0.00 (0.00-0.00)	0.00 (0.00-0.00)	0.00 (0.00-0.01)	0.01 (0.00-0.01)	0.01 (0.00-0.03)	0.00 (0.00-0.01)	-	-	
8	1388	0.13 (0.09-0.17)	0.11 (0.09-0.14)	0.11 (0.09-0.13)	0.11 (0.09-0.13)	0.08 (0.07-0.10)	0.03 (0.01-0.05)	0.02 (0.00-0.04)	-	
9	1608	0.27 (0.22-0.33)	0.23 (0.20-0.26)	0.10 (0.09-0.11)	0.10 (0.09-0.11)	0.05 (0.03-0.06)	0.01 (0.00-0.02)	0.00 (0.00-0.00)	-	
**Healthy diet score**[#]										<0.001
<56.9	4255	0.52 (0.45-0.59)	0.48 (0.45-0.51)	0.40 (0.37-0.43)	0.33 (0.31-0.36)	0.28 (0.25-0.31)	0.23 (0.19-0.26)	0.09 (0.05-0.12)	-	
56.9-69.3	4521	0.47 (0.40-0.54)	0.41 (0.37-0.44)	0.37 (0.34-0.39)	0.33 (0.30-0.35)	0.30 (0.26-0.33)	0.26 (0.22-0.30)	0.16 (0.11-0.21)	-	

© 2019 Han L et al. *JAMA Network Open.*

≥69.3	4830	-	0.10 (0.08-0.12)	0.22 (0.19-0.24)	0.32 (0.30-0.35)	0.41 (0.37-0.45)	0.41 (0.37-0.45)	0.74 (0.68-0.80)	1.00 (1.00-1.00)	
**Total serum cholesterol , mg/dL**										<0.001
≥240	2638	0.46 (0.39-0.53)	0.36 (0.33-0.39)	0.29 (0.26-0.32)	0.18 (0.15-0.20)	0.06 (0.04-0.08)	0.03 (0.01-0.04)	0.00 (0.00-0.01)	-	
200-239 or treated to goal	4115	0.53 (0.46-0.60)	0.52 (0.49-0.55)	0.41 (0.38-0.44)	0.31 (0.29-0.34)	0.18 (0.15-0.21)	0.09 (0.07-0.11)	0.02 (0.00-0.03)	-	
< 200 (untreated) **	6853	-	0.11 (0.08-0.14)	0.28 (0.25-0.31)	0.50 (0.47-0.53)	0.74 (0.71-0.78)	0.87 (0.84-0.89)	0.97 (0.95-0.98)	1.00 (1.00-1.00)	
**Blood pressure**										<0.001
SBP ≥130 or DBP ≥80 mm Hg	3016	0.44 (0.38-0.50)	0.36 (0.32-0.39)	0.29 (0.26-0.31)	0.20 (0.18-0.22)	0.10 (0.08-0.12)	0.04 (0.02-0.06)	0.02 (0.00-0.04)	-	
SBP 120–129 or DBP <80 mmHg or treated to goal	5120	0.55 (0.49-0.61)	0.55 (0.52-0.59)	0.47 (0.43-0.50)	0.35 (0.33-0.37)	0.22 (0.20-0.25)	0.09 (0.07-0.11)	0.02 (0.00-0.04)	-	
<120/80(untreated**	5470	-	0.07 (0.06-0.09)	0.23 (0.20-0.26)	0.43 (0.41-0.46)	0.66 (0.63-0.69)	0.86 (0.83-0.89)	0.95 (0.92-0.98)	1.00 (1.00-1.00)	
**Fasting blood glucose, mg/dL**††										<0.001
≥126	551	0.26 (0.17-0.34)	0.14 (0.11-0.17)	0.06 (0.04-0.08)	0.02 (0.01-0.03)	0.00 (0.00-0.00)	-	-	-	
100-<125	1582	0.73 (0.65-0.82)	0.39 (0.35-0.44)	0.22 (0.19-0.25)	0.10 (0.07-0.13)	0.05 (0.03-0.07)	0.03 (0.01-0.05)	-	-	
<100	3451	-	0.46 (0.40-0.51)	0.71 (0.67-0.74)	0.86 (0.83-0.90)	0.94 (0.92-0.96)	0.96 (0.94-0.98)	1.00 (1.00-1.00)	1.00 (1.00-1.00)	

Abbreviations:BMI-WHR, body mass index-waist-hip ratio; NHANES, National Health and Nutrition Examination Survey.

© 2019 Han L et al. *JAMA Network Open.*

[*]. All nonpregnant participants older than 20 years with available revised Life's Simple 7 metrics were included.

[†]. Weighted prevalence and 95% CIs

[‡]. Continuous variables or categorical variables across Life's Simple 7 metrics were performed using one-way ANOVA or Chi-square or nonparametric tests, as appropriate.

[§]. See the definition of physical activity from table 1.

[‖]. The classification of BMI combined with WHR was based on the reference 2 in the paper.

1 for BMI<=24.9, WHR<0.9 and sex=male; BMI<=24.9, WHR<0.8 and sex=female. 2 for BMI<=24.9, WHR<1 and sex=male; BMI<=24.9, WHR<0.92 and sex=female. 3 for BMI<=24.9, WHR>1 and sex=male; BMI<=24.9, WHR>0.92 and sex=female. 4 for BMI<=29.9, WHR<0.9 and sex=male; BMI<=29.9, WHR<0.8 and sex=female. 5 for BMI<=29.9, WHR<1 and sex=male; BMI<=29.9, WHR<0.92 and sex=female. 6 for BMI<=29.9, WHR>1 and sex=male; BMI<=29.9, WHR>0.92 and sex=female. 7 for BMI≥30, WHR<0.9 and sex=male; BMI≥30, WHR<0.8 and sex=female. 8 for BMI≥30, WHR<1 and sex=male; BMI≥30, WHR<0.92 and sex=female. 9 for BMI≥30, WHR>1 and sex=male; BMI≥30, WHR>0.92 and sex=female.

[#]. The healthy diet score was calculated based on the healthy eating index-2010. The tertiles of HEI was classified as revised ideal, intermediate and poor, respectively.

[**]. Untreated value.

[††]. Fasting glucose was only available in a sub-group of NHANES III (1988-1994).

© 2019 Han L et al. *JAMA Network Open.*

eTable 3: Characteristics of US Adults—NHANES III (1988-1994), 1999-2004, 2005-2010, 2011-2016

Characteristics	NHANES III(1988-1994)*		NHANES 1999-2004*		NHANES 2005-2010*		NHANES 2011-2016*	
	No.	Prevalence,% (95% CI)	No.	Prevalence,% (95% CI)	No.	Prevalence,% (95% CI)	No.	Prevalence,% (95% CI)
Age, mean (95% CI), y	13606	43.4(42.6-44.3)	6360	44.7(44.1-45.2)	10618	44.6(43.9-45.2)	10773	44.5(43.8-45.2)
Sex								
male	6539	49.1(48.2-50.1)	3128	48.9(47.6-50.1)	5220	48.5(47.7-49.2)	5093	47.0(46.0-48.0)
female	7067	50.8(49.8-51.7)	3232	51.1(49.8-52.3)	5398	51.5(50.7-52.2)	5680	53.0(51.9-53.9)
Race/ethnicity								
Non-Hispanic White	5263	45.4(41.8-49.0)	4615	53.4(48.2-58.4)	5069	51.9(46.5-57.1)	3855	38.5(33.5-43.3)
Non-Hispanic Black	3910	25.5(23.0-28.0)	1788	17.3(13.7-20.7)	2011	18.0(15.1-20.7)	2363	21.6(17.7-25.5)
Mexican American	3862	25.0(23.0-27.0)	2213	22.2(16.6-27.8)	2118	18.1(14.5-21.7)	1561	13.6(10.0-17.1)
Other	571	3.91(2.63-5.18)	733	7.10(4.87-9.34)	1420	12.0(9.48-14.6)	2994	26.3(23.3-29.2)

Abbreviation: NHANES, National Health and Nutrition Examination Survey.

*. Age-standardized for the entire population by the direct method to the US 2000 Census population using the age groups 20-29, 30-39, 40-49, 50-59, 60-69, 70-79, and 80 years or older.

© 2019 Han L et al. *JAMA Network Open.*

eTable 4: Adjusted HRs (95% CIs) for All-Cause and Cause-Specific Mortality by Numbers of revised ideal Life's Simple 7 Metrics and Age Groups, NHANES III (1988-1994) Linked Mortality File-2011

Characteristics[*]	Numbers of revised ideal Life's Simple 7 metrics					p-value for trends[†]
	0-1	2	3	4	5-7	
**All-cause mortality**						
**Age <60 years**						
All deaths	295	258	210	131	68	
Number of participants	1211	1916	2347	2176	1690	
Total person years	22039	36384	45265	42549	33107	
Crude	1 [Reference]	0.47 (0.38-0.57)	0.36 (0.26-0.49)	0.22 (0.16-0.30)	0.14 (0.09-0.21)	<0.001
Adjusted for sex and race-ethnicity	1 [Reference]	0.47 (0.39-0.57)	0.37 (0.27-0.50)	0.22 (0.16-0.31)	0.14 (0.09-0.22)	<0.001
**Age≥60 years**						
All deaths	636	684	461	201	71	
Number of participants	861	945	721	323	109	
Total person years	10743	12249	9967	4564	1506	
Crude	1 [Reference]	1.01(0.83-1.23)	0.70 (0.58-0.84)	0.60 (0.47-0.77)	0.56 (0.40-0.78)	<0.001
Adjusted for sex and race-ethnicity	1 [Reference]	1.02 (0.84-1.25)	0.70 (0.58-0.85)	0.61 (0.47-0.78)	0.57 (0.41-0.80)	<0.001
**CVD mortality**						
**Age < 60 years**						
CVD deaths	71	57	32	18	6	
Number of participants	987	1717	2169	2063	1628	
Total person years	22039	36384	45265	42549	33107	
Crude	1	0.48	0.29	0.12	0.03	<0.001

© 2019 Han L et al. *JAMA Network Open.*

	[Reference]	(0.32-0.72)	(0.16-0.51)	(0.03-0.37)	(0.01-0.10)	
Adjusted for sex and race-ethnicity	1 [Reference]	0.49 (0.32-0.75)	0.30 (0.17-0.53)	0.12 (0.04-0.38)	0.04 (0.01-0.12)	<0.001
**Age≥60 years**						
CVD deaths	197	235	158	60	23	
Number of participants	422	496	418	182	61	
Total person years	10743	12249	9967	4564	1506	
Crude	1 [Reference]	1.15 (0.85-1.56)	0.73 (0.53-1.01)	0.49 (0.29-0.82)	0.57 (0.32-1.02)	<0.001
Adjusted for sex and race-ethnicity	1 [Reference]	1.17 (0.86-1.59)	0.73 (0.53-1.00)	0.49 (0.29-0.82)	0.59 (0.32-1.06)	<0.001
**Cancer mortality**						
**Age < 60 years**						
Cancer deaths	84	79	59	41	19	
Number of participants	1000	1737	2196	2086	1641	
Total person years	22039	36384	45265	42549	33107	
Crude	1 [Reference]	0.51 (0.36-0.72)	0.29 (0.17-0.51)	0.22 (0.12-0.40)	0.13 (0.07-0.24)	<0.001
Adjusted for sex and race-ethnicity	1 [Reference]	0.48 (0.34-0.68)	0.27 (0.16-0.47)	0.20 (0.11-0.36)	0.11 (0.06-0.20)	<0.001
**Age≥60 years**						
Cancer deaths	138	129	89	40	17	
Number of participants	363	390	349	162	55	
Total person years	10743	12249	9967	4564	1506	
Crude	1 [Reference]	1.02 (0.62-1.68)	0.60 (0.42-0.87)	0.58 (0.34-1.00)	0.47 (0.25-0.90)	<0.001

© 2019 Han L et al. *JAMA Network Open.*

Adjusted for sex and race-ethnicity	1 [Reference]	1.05 (0.64-1.73)	0.64 (0.44-0.94)	0.60 (0.35-1.04)	0.52 (0.27-0.98)	<0.001

Abbreviations: BMI-WHR, body mass index-waist-hip ratio; NHANES, National Health and Nutrition Examination Survey; CVD, cardiovascular disease; NA, not available.

[*]. All nonpregnant participants older than 20 years with available revised Life's Simple 7 metrics were included.

[†]. Trends for numbers of revised ideal Life's Simple 7 metrics were analyzed by logistic regression model adjusted for age, sex, and race/ethnicity.

© 2019 Han L et al. *JAMA Network Open.*

eTable 5: Adjusted HRs (95% CIs) for All-Cause and Cause-Specific Mortality by Numbers of revised ideal Life's Simple 7 Metrics and Sex, NHANES III (1988-1994) Linked Mortality File-2011

**Characteristics [a]**	**Numbers of revised ideal Life's Simple 7 metrics**					**p-value for trends[†]**
	**0-1**	**2**	**3**	**4**	**5-7**	
**All-cause mortality**						
**Male**						
All deaths	538	498	326	142	84	
Number of participants	1245	1508	1416	1022	723	
Total person years	20025	25644	25306	19317	13768	
Crude	1 [Reference]	0.60 (0.47-0.76)	0.41 (0.32-0.52)	0.23 (0.19-0.29)	0.22 (0.16-0.31)	<0.001
Adjusted for age and race-ethnicity	1 [Reference]	0.72 (0.60-0.88)	0.60 (0.46-0.79)	0.48 (0.38-0.62)	0.68 (0.49-0.96)	<0.001
**Female**						
All deaths	393	444	345	190	55	
Number of participants	827	1353	1652	1477	1076	
Total person years	12756	22989	29925	27797	20846	
Crude	1 [Reference]	0.60 (0.51-0.71)	0.39 (0.33-0.47)	0.20 (0.16-0.27)	0.07 (0.05-0.10)	<0.001
Adjusted for age and race-ethnicity	1 [Reference]	0.82 (0.68-0.98)	0.60 (0.49-0.74)	0.50 (0.39-0.65)	0.32 (0.22-0.47)	<0.001
**CVD mortality**						
**Male**						
CVD deaths	141	147	91	37	17	
Number of participants	848	1157	1181	917	656	
Total person years	20025	25644	25306	19317	13768	
Crude	1	0.64	0.48	0.14	0.08	<0.001

© 2019 Han L et al. *JAMA Network Open.*

	[Reference]	(0.44-0.93)	(0.30-0.75)	(0.08-0.25)	(0.04-0.16)	
Adjusted for age and race-ethnicity	1<br>[Reference]	0.84<br>(0.57-1.22)	0.67<br>(0.40-1.11)	0.33<br>(0.19-0.58)	0.33<br>(0.16-0.69)	<0.001
**Female**						
CVD deaths	127	147	99	41	12	
Number of participants	561	1056	1406	1328	1033	
Total person years	12756	22989	29925	27797	20846	
Crude	1<br>[Reference]	0.56<br>(0.39-0.81)	0.30<br>(0.22-0.42)	0.14<br>(0.07-0.25)	0.06<br>(0.03-0.12)	<0.001
Adjusted for age and race-ethnicity	1<br>[Reference]	0.92<br>(0.61-1.37)	0.51<br>(0.35-0.75)	0.47<br>(0.24-0.90)	0.40<br>(0.20-0.81)	<0.001
**Cancer mortality**						
**Male**						
Cancer deaths	138	117	73	37	20	
Number of participants	845	1127	1163	917	659	
Total person years	20025	25644	25306	19317	13768	
Crude	1<br>[Reference]	0.46<br>(0.29-0.73)	0.28<br>(0.17-0.44)	0.25<br>(0.14-0.46)	0.19<br>(0.10-0.37)	<0.001
Adjusted for age and race-ethnicity	1<br>[Reference]	0.64<br>(0.41-1.00)	0.40<br>(0.25-0.65)	0.55<br>(0.31-0.98)	0.73<br>(0.39-1.37)	<0.001
**Female**						
Cancer deaths	84	91	75	44	16	
Number of participants	518	1000	1382	1331	1037	
Total person years	12756	22989	29925	27797	20846	
Crude	1<br>[Reference]	0.66<br>(0.47-0.94)	0.40<br>(0.25-0.65)	0.20<br>(0.11-0.34)	0.07<br>(0.03-0.16)	<0.001

© 2019 Han L et al. *JAMA Network Open*.

Adjusted for age and race-ethnicity	1 [Reference]	0.97 (0.65-1.13)	0.62 (0.38-1.03)	0.46 (0.26-0.79)	0.23 (0.09-0.58)	<0.001

Abbreviations: BMI-WHR, body mass index-waist-hip ratio; NHANES, National Health and Nutrition Examination Survey; CVD, cardiovascular disease; NA, not available.

*. All nonpregnant participants older than 20 years with available revised Life's Simple 7 metrics were included.

†. Trends for numbers of revised ideal Life's Simple 7 metrics were analyzed by logistic regression model adjusted for age, sex, and race/ethnicity.

© 2019 Han L et al. *JAMA Network Open.*

eTable 6: Adjusted HRs (95% CIs) for All-Cause and Cause-Specific Mortality by Numbers of revised ideal Life's Simple 7 Metrics and Race/Ethnicity, NHANES III (1988-1994) Linked Mortality File-2011

Characteristics[*]	Numbers of revised ideal Life's Simple 7 metrics					p-value for trends[†]
	0-1	2	3	4	5-7	
**All-cause mortality**						
**Non-Hispanic White**						
All deaths	392	436	358	182	69	
Number of participants	758	1104	1175	896	660	
Total person years	11408	18020	20156	16300	12610	
Crude	1 [Reference]	0.58 (0.50-0.67)	0.42 (0.36-0.49)	0.23 (0.19-0.28)	0.13 (0.10-0.18)	<0.001
Adjusted for age and sex	1 [Reference]	0.74 (0.63-0.86)	0.58 (0.47-0.72)	0.47 (0.37-0.60)	0.48 (0.35-0.66)	<0.001
**Non-Hispanic Black**						
All deaths	287	275	171	71	37	
Number of participants	680	859	882	684	489	
Total person years	10946	14544	15974	13092	9383	
Crude	1 [Reference]	0.72 (0.59-0.88)	0.39 (0.32-0.47)	0.20 (0.15-0.26)	0.14 (0.10-0.20)	<0.001
Adjusted for age and sex	1 [Reference]	0.90 (0.73-1.12)	0.71 (0.57-0.89)	0.55 (0.42-0.70)	0.59 (0.42-0.81)	0.001
**Mexican American**						
All deaths	237	202	126	63	28	
Number of participants	578	795	888	780	548	
Total person years	9494	14274	16810	15144	10702	
Crude	1 [Reference]	0.57 (0.44-0.73)	0.31 (0.24-0.41)	0.21 (0.15-0.29)	0.13 (0.07-0.22)	<0.001

© 2019 Han L et al. *JAMA Network Open.*

Adjusted for age and sex	1 [Reference]	0.83 (0.66-1.04)	0.64 (0.47-0.85)	0.67 (0.48-0.94)	0.58 (0.31-1.11)	<0.001
**CVD mortality**						
**Non-Hispanic White**						
CVD deaths	112	140	111	45	15	
Number of participants	478	808	928	759	606	
Total person years	11408	18020	20156	16300	12610	
Crude	1 [Reference]	0.60 (0.47-0.77)	0.43 (0.30-0.61)	0.15 (0.09-0.24)	0.07 (0.04-0.12)	<0.001
Adjusted for age and sex	1 [Reference]	0.84 (0.63-1.14)	0.56 (0.37-0.84)	0.36 (0.21-0.61)	0.35 (0.22-0.56)	<0.001
**Non-Hispanic Black**						
CVD deaths	80	80	39	17	6	
Number of participants	473	664	750	630	458	
Total person years	10946	14544	15974	13092	9383	
Crude	1 [Reference]	0.71 (0.50-1.00)	0.31 (0.20-0.47)	0.15 (0.09-0.25)	0.08 (0.03-0.19)	<0.001
Adjusted for age and sex	1 [Reference]	0.97 (0.68-1.38)	0.71 (0.48-1.06)	0.61 (0.36-1.02)	0.62 (0.27-1.39)	0.001
**Mexican American**						
CVD deaths	70	68	38	11	4	
Number of participants	411	661	800	728	524	
Total person years	9494	14274	16810	15144	10702	
Crude	1 [Reference]	0.61 (0.39-0.96)	0.23 (0.14-0.39)	0.06 (0.02-0.18)	0.04 (0.01-0.21)	<0.001
Adjusted for age and sex	1 [Reference]	0.90	0.54	0.28	0.25	<0.001

© 2019 Han L et al. *JAMA Network Open.*

		(0.61-1.32)	(0.31-0.93)	(0.10-0.78)	(0.04-1.42)	
**Cancer mortality**						
**Non-Hispanic White**						
Cancer deaths	95	95	68	48	17	
Number of participants	461	763	885	762	608	
Total person years	11408	18020	20156	16300	12610	
Crude	1 [Reference]	0.54 (0.41-0.71)	0.33 (0.23-0.48)	0.26 (0.16-0.42)	0.12 (0.07-0.22)	<0.001
Adjusted for age and sex	1 [Reference]	0.78 (0.56-1.08)	0.46 (0.30-0.70)	0.55 (0.33-0.93)	0.44 (0.24-0.83)	<0.001
**Non-Hispanic Black**						
Cancer deaths	80	70	49	15	8	
Number of participants	473	654	760	628	460	
Total person years	10946	14544	15974	13092	9383	
Crude	1 [Reference]	0.60 (0.45-0.80)	0.33 (0.22-0.48)	0.11 (0.06-0.19)	0.09 (0.03-0.22)	<0.001
Adjusted for age and sex	1 [Reference]	0.78 (0.55-1.09)	0.64 (0.42-0.97)	0.33 (0.20-0.55)	0.44 (0.17-1.11)	0.144
**Mexican American**						
Cancer deaths	45	38	24	17	10	
Number of participants	386	631	786	734	530	
Total person years	9494	14274	16810	15144	10702	
Crude	1 [Reference]	0.54 (0.26-1.11)	0.30 (0.19-0.45)	0.35 (0.19-0.65)	0.22 (0.10-0.47)	<0.001
Adjusted for age and sex	1 [Reference]	0.76 (0.36-1.63)	0.65 (0.40-1.05)	1.37 (0.70-2.67)	1.44 (0.55-3.77)	0.386

© 2019 Han L et al. *JAMA Network Open.*

Abbreviations: NHANES, National Health and Nutrition Examination Survey; CVD, cardiovascular disease; NA, not available.

[*] All nonpregnant participants older than 20 years with available revised Life's Simple 7 metrics were included.

[†] Trends for numbers of revised ideal Life's Simple 7 metrics were analyzed by logistic regression model adjusted for age, sex, and race/ethnicity.

© 2019 Han L et al. *JAMA Network Open.*

eTable 7: Adjusted HRs (95% CIs) for All-Cause and Cause-Specific Mortality by Numbers of revised ideal Life's Simple 7 Metrics and Educational Attainment, NHANES III (1988-1994) Linked Mortality File-2011

**Characteristics***	**Numbers of revised ideal Life's Simple 7 metrics**					**p-value for trends†**
	**0-1**	**2**	**3**	**4**	**5-7**	
**All-cause mortality**						
**Education<12 years**						
All deaths	583	527	335	170	54	
Number of participants	1093	1249	1143	792	424	
Total person years	16430	20103	19726	14295	7932	
Crude	1 [Reference]	0.70 (0.58-0.86)	0.51 (0.41-0.64)	0.34 (0.27-0.43)	0.15 (0.10-0.23)	<0.001
Adjusted for age, sex and race-ethnicity	1 [Reference]	0.85 (0.69-1.05)	0.70 (0.54-0.91)	0.67 (0.52-0.87)	0.56 (0.39-0.81)	<0.001
**Education≥12 years**						
All deaths	348	415	336	162	85	
Number of participants	979	1612	1925	1707	1375	
Total person years	16351	28530	35505	32819	26682	
Crude	1 [Reference]	0.62 (0.51-0.74)	0.41 (0.35-0.49)	0.23 (0.18-0.29)	0.15 (0.11-0.20)	<0.001
Adjusted for age, sex and race-ethnicity	1 [Reference]	0.75 (0.64-0.88)	0.58 (0.48-0.70)	0.46 (0.35-0.61)	0.47 (0.34-0.65)	<0.001
**CVD mortality**						
**Education<12 years**						
CVD deaths	177	173	101	44	11	
Number of participants	687	895	909	666	381	

© 2019 Han L et al. *JAMA Network Open.*

Total person years	16430	20103	19726	14295	7932	
Crude	1<br>[Reference]	0.69<br>(0.46-1.03)	0.40<br>(0.24-0.65)	0.23<br>(0.14-0.37)	0.10<br>(0.04-0.22)	<0.001
Adjusted for age, sex and race-ethnicity	1<br>[Reference]	0.93<br>(0.59-1.46)	0.59<br>(0.32-1.09)	0.62<br>(0.41-0.94)	0.63<br>(0.31-1.29)	<0.001
**Education≥12 years**						
CVD deaths	91	121	89	34	18	
Number of participants	722	1318	1678	1579	1308	
Total person years	16351	28530	35505	32819	26682	
Crude	1<br>[Reference]	0.67<br>(0.50-0.91)	0.47<br>(0.33-0.68)	0.16<br>(0.08-0.21)	0.09<br>(0.05-0.15)	<0.001
Adjusted for age, sex and race-ethnicity	1<br>[Reference]	0.88<br>(0.66-1.18)	0.61<br>(0.42-0.88)	0.38<br>(0.19-0.75)	0.36<br>(0.23-0.58)	<0.001
**Cancer mortality**						
**Education<12 years**						
Cancer deaths	136	110	69	31	15	
Number of participants	646	832	877	653	385	
Total person years	16430	20103	19726	14295	7932	
Crude	1<br>[Reference]	0.55<br>(0.36-0.85)	0.39<br>(0.22-0.68)	0.23<br>(0.13-0.41)	0.09<br>(0.04-0.22)	<0.001
Adjusted for age, sex and race-ethnicity	1<br>[Reference]	0.78<br>(0.47-1.31)	0.58<br>(0.32-1.08)	0.64<br>(0.33-1.23)	0.44<br>(0.17-1.12)	<0.001
**Education≥12 years**						
Cancer deaths	86	98	79	50	21	
Number of participants	717	1295	1668	1595	1311	
Total person years	16351	28530	35505	32819	26682	

© 2019 Han L et al. *JAMA Network Open.*

Crude	1 [Reference]	0.63 (0.45-0.87)	0.37 (0.25-0.53)	0.27 (0.16-0.44)	0.14 (0.08-0.25)	<0.001
Adjusted for age, sex and race-ethnicity	1 [Reference]	0.84 (0.60-1.17)	0.51 (0.36-0.74)	0.52 (0.30-0.89)	0.45 (0.25-0.79)	<0.001

Abbreviations: NHANES, National Health and Nutrition Examination Survey; CVD, cardiovascular disease; NA, not available.

[*]. All nonpregnant participants older than 20 years with available revised Life's Simple 7 metrics were included.

[†]. Trends for numbers of revised ideal Life's Simple 7 metrics were analyzed by logistic regression model adjusted for age, sex, and race/ethnicity.

© 2019 Han L et al. *JAMA Network Open.*

eTable 8: Adjusted HRs (95% CIs) for All-Cause and Cause-Specific Mortality by Numbers of revised ideal Life's Simple 7 Metrics and Alcohol Groups, NHANES III (1988-1994) Linked Mortality File-2011

Characteristics*	Numbers of revised ideal Life's Simple 7 metrics					p-value for trends†
	0-1	2	3	4	5-7	
**All-cause mortality**						
**0 drinks/week**						
All deaths	668	658	455	235	91	
Number of participants	1287	1748	1830	1550	1130	
Total person years	19336.75	29008.17	32212.33	28902.17	21628	
Crude	1	0.56	0.40	0.20	0.10	<0.001
	[Reference]	(0.48-0.66)	(0.35-0.48)	(0.16-0.26)	(0.08-0.15)	
Adjusted for age, sex and race-ethnicity	1	0.71	0.56	0.44	0.37	<0.001
	[Reference]	(0.61-0.82)	(0.45-0.71)	(0.34-0.55)	(0.27-0.52)	
**<3 drinks/week**						
All deaths	111	86	90	49	12	
Number of participants	345	488	588	481	361	
Total person years	5901.92	8919.5	11045.42	9241.25	7174.5	
Crude	1	0.48	0.56	0.39	0.13	<0.001
	[Reference]	(0.33-0.73)	(0.36-0.90)	(0.24-0.66)	(0.05-0.34)	
Adjusted for age, sex and race-ethnicity	1	0.58	0.79	0.67	0.37	<0.001
	[Reference]	(0.38-0.87)	(0.48-1.30)	(0.40-1.15)	(0.14-0.95)	
**⩾3 drinks/week**						
All deaths	148	189	125	46	34	
Number of participants	434	612	648	460	305	

© 2019 Han L et al. *JAMA Network Open.*

Total person years	7446.08	10504.42	11942	8821.5	5779.08	
Crude	1	0.91	0.42	0.24	0.25	<0.001
	[Reference]	(0.65-1.28)	(0.31-0.59)	(0.16-0.37)	(0.15-0.46)	
Adjusted for age, sex and race-ethnicity	1	1.09	0.6	0.55	0.73	<0.001
	[Reference]	(0.76-1.58)	(0.41-0.88)	(0.34-0.89)	(0.41-1.31)	
**CVD mortality**						
**0 drinks/week**						
CVD deaths	204	205	133	59	23	
Number of participants	823	1295	1508	1374	1062	
Total person years	19336.75	29008.17	32212.33	28902.17	21628	
Crude	1	0.51	0.4	0.16	0.08	<0.001
	[Reference]	(0.37-0.69)	(0.27-0.58)	(0.09-0.27)	(0.04-0.13)	
Adjusted for age, sex and race-ethnicity	1	0.71	0.54	0.39	0.33	<0.001
	[Reference]	(0.53-0.96)	(0.33-0.89)	(0.23-0.68)	(0.20-0.54)	
**<3 drinks/week**						
CVD deaths	24	21	20	14	3	
Number of participants	258	423	518	446	352	
Total person years	5901.92	8919.5	11045.42	9241.25	7174.5	
Crude	1	0.32	0.33	0.31	0.12	<0.001
	[Reference]	(0.13-0.84)	(0.09-1.14)	(0.11-0.86)	(0.03-0.57)	
Adjusted for age, sex and race-ethnicity	1	0.44	0.54	0.64	0.48	<0.001
	[Reference]	(0.16-1.19)	(0.15-1.94)	(0.20-2.03)	(0.09-2.58)	
**⩾3 drinks/week**						
CVD deaths	39	64	36	5	3	

© 2019 Han L et al. *JAMA Network Open.*

Number of participants	325	487	559	419	274	
Total person years	7446.08	10504.42	11942	8821.5	5779.08	
Crude	1	1.36	0.46	0.04	0.05	<0.001
	[Reference]	(0.87-2.12)	(0.25-0.85)	(0.01-0.15)	(0.01-0.27)	
Adjusted for age, sex and race-ethnicity	1	1.89	0.69	0.13	0.27	<0.001
	[Reference]	(1.04-3.44)	(0.37-1.29)	(0.04-0.49)	(0.05-1.54)	
**Cancer mortality**						
**0 drinks/week**						
Cancer deaths	151	135	99	55	23	
Number of participants	770	1225	1474	1370	1062	
Total person years	19336.75	29008.17	32212.33	28902.17	21628	
Crude	1	0.53	0.34	0.21	0.07	<0.001
	[Reference]	(0.40-0.70)	(0.22-0.52)	(0.13-0.35)	(0.03-0.14)	
Adjusted for age, sex and race-ethnicity	1	0.75	0.47	0.48	0.23	<0.001
	[Reference]	(0.54-1.05)	(0.29-0.75)	(0.29-0.77)	(0.1-0.52)	
**<3 drinks/week**						
Cancer deaths	28	23	16	11	3	
Number of participants	262	425	514	443	352	
Total person years	5901.92	8919.5	11045.42	9241.25	7174.5	
Crude	1	0.7	0.42	0.29	0.1	<0.001
	[Reference]	(0.28-1.77)	(0.18-0.97)	(0.11-0.73)	(0.02-0.59)	
Adjusted for age, sex and race-ethnicity	1	0.91	0.69	0.55	0.33	<0.001
	[Reference]	(0.36-2.34)	(0.27-1.75)	(0.18-1.66)	(0.04-2.45)	
**⩾3 drinks/week**						

© 2019 Han L et al. *JAMA Network Open.*

Cancer deaths	42	48	33	15	10	
Number of participants	328	471	556	429	281	
Total person years	7446.08	10504.42	11942	8821.5	5779.08	
Crude	1	0.58	0.35	0.24	0.3	<0.001
	[Reference]	(0.33-1.01)	(0.19-0.61)	(0.11-0.53)	(0.12-0.72)	
Adjusted for age, sex and race-ethnicity	1	0.78	0.51	0.54	0.92	<0.001
	[Reference]	(0.42-1.45)	(0.28-0.93)	(0.21-1.36)	(0.35-2.40)	

Abbreviations: BMI-WHR, body mass index-waist-hip ratio; NHANES, National Health and Nutrition Examination Survey; CVD, cardiovascular disease;

NA, not available.

[*]. All nonpregnant participants older than 20 years with available revised Life's Simple 7 metrics were included.

[†].Trends for numbers of revised ideal Life's Simple 7 metrics were analyzed by logistic regression model adjusted for age, sex, and race/ethnicity.

© 2019 Han L et al. *JAMA Network Open*.

eFigure 1: Adjusted Hazard Ratios and Population Attributable Fractions for All-Cause and Cause-Specific Mortality by Revised Life's Simple 7 metrics

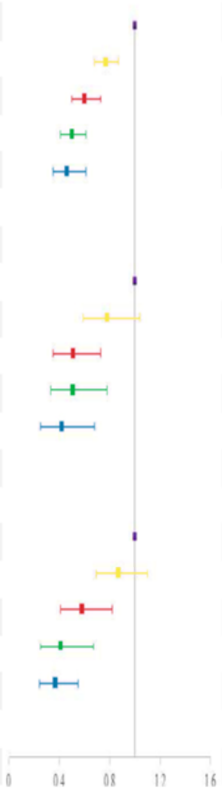

	Age, sex and race adjusted	Curde HRs (95% CIs)	No. of participants	Cases	Total person-years	Cases/Pys (/1000)	P Value for Trends	Adjusted PAF (95% CI)
All-cause mortality							<0.001	68.4(63.1-73.0)
0-1	1	1	2072	931	32782	28.40		
2	0.77(0.68-0.87)	0.61(0.54-0.70)	2861	942	48633	19.37		
3	0.60(0.50-0.73)	0.41(0.36-0.47)	3068	671	55232	12.15		
4	0.50(0.41-0.61)	0.23(0.19-0.27)	2499	332	47114	7.05		
5-7	0.46(0.35-0.61)	0.13(0.10-0.17)	1799	139	34614	4.02		
Cancer mortality							<0.001	69.9(31.2-88.6)
0-1	1	1	1363	222	32782	6.77		
2	0.78(0.59-1.04)	0.56(0.44-0.71)	2127	208	48633	4.28		
3	0.51(0.35-0.73)	0.35(0.25-0.48)	2545	148	55232	2.68		
4	0.51(0.33-0.78)	0.23(0.15-0.34)	2248	81	47114	1.72		
5-7	0.42(0.25-0.68)	0.11(0.07-0.18)	1696	36	34614	1.04		
CVD mortality							<0.001	48.9(28.7-63.3)
0-1	1	1	1409	268	32782	8.18		
2	0.87(0.69-1.10)	0.63(0.51-0.77)	2213	294	48633	6.05		
3	0.58(0.41-0.82)	0.40(0.30-0.53)	2587	190	55232	3.44		
4	0.41(0.25-0.67)	0.15(0.09-0.24)	2245	78	47114	1.66		
5-7	0.37(0.24-0.55)	0.07(0.05-0.11)	1689	29	34614	0.84		

PAF was adjusted for age and sex.

© 2019 Han L et al. *JAMA Network Open.*

eFigure 2: The adjusted Hazard Ratios and Population Attributable Fractions for All-Cause and Cause-Specific Mortality by each revised Life's Simple 7 Metrics and that recommended by AHA

	Fully adjusted in the revised LS7 metrics[‡]	Adjusted PAF(95% CI) in the revised LS7 metrics[§]
**All-cause mortality**		
**BMI-WHR[*]**		16.61 (5.2-26.6)
Normal weight with central obesity	1	
BMI≤29.9 kg/m2 but without central obesit	0.61(0.44-0.85)	
**Healthy diet score[†]**		8.0 (4.7-11.1)
<56.9	1	
56.9-69.3	0.86 (0.73-1.01)	
≥69.3	0.81 (0.70-0.93)	
**Blood pressure, mm Hge**		37.4 (32.8-42.5)
SBP ≥130 or DBP ≥80 mmHg	1	
SBP 120–129 or DBP <80 mmHg or treatc goal	0.99 (0.85-1.16)	
<120/80 mmHg (untreated)	0.73 (0.61-0.88)	
**CVD mortality**		
**BMI-WHR[*]**		NA
Normal weight with central obesity	1	
BMI≤29.9 kg/m2 but without central obesit	0.44(0.23-0.83)	
**Healthy diet score[†]**		NA
<56.9	1	
56.9-69.3	0.86 (0.60-1.24)	
≥69.3	0.91 (0.72-1.14)	
**Blood pressure, mm Hge**		36.7 (26.8-28.9)
SBP ≥130 or DBP ≥80 mmHg	1	
SBP 120–129 or DBP <80 mmHg or treatc goal	0.83 (0.64-1.08)	
<120/80 mmHg (untreated)	0.56 (0.38-0.81)	
**Cancer mortality**		
**BMI-WHR[*]**		NA
Normal weight with central obesity	1	
BMI≤29.9 kg/m2 but without central obesit	0.70(0.38-1.29)	
**Healthy diet score[†]**		11.67(2.0-20.4)
<56.9	1	
56.9-69.3	0.83 (0.59-1.18)	
≥69.3	0.72 (0.51-1.00)	
**Blood pressure, mm Hg**		47.5(38.2-57.3)
SBP ≥130 or DBP ≥80 mmHg	1	
SBP 120–129 or DBP <80 mmHg or treatc goal	0.82 (0.56-1.19)	
<120/80 mmHg (untreated)[f]	0.59 (0.41-0.84)	

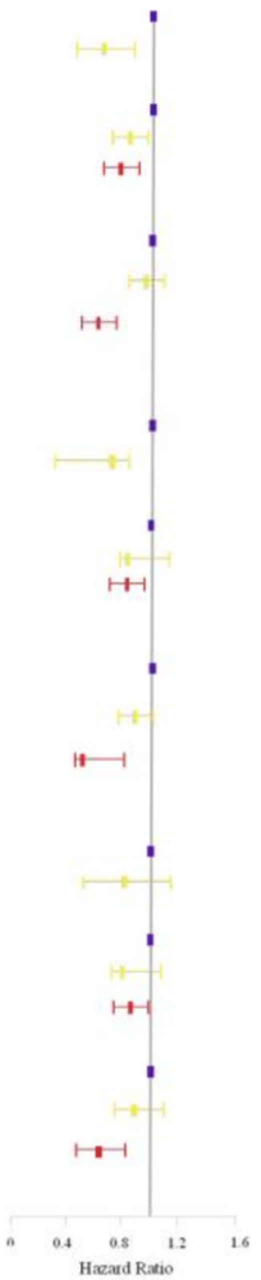

	Fully adjusted in the previous paper[ǁ]	Adjusted PAF(95% CI) in the previous paper[#]
**All-cause mortality**		
**BMI, kg/m²**		NA
≥25	1	
<25	1.04 (0.93-1.17)	
**Healthy diet score[¥]**		1.8 (0.0-15.2)
< 2 components	1	
≥ 2 components	0.94 (0.83-1.07)	
**Blood pressure, mm Hg**		30.4 (19.4-40.6)
≥ 120/ ≥80	1	
< 120/ <80 (untreated)[f]	0.81 (0.68-0.95)	
**CVD mortality**		
**BMI, kg/m²**		NA
≥25	1	
<25	1.05 (0.88-1.25)	
**Healthy diet score[¥]**		13.2 (3.5-29.2)
< 2 components	1	
≥ 2 components	0.82 (0.67-1.02)	
**Blood pressure, mm Hg**		40.6 (24.5-54.6)
≥ 120 /≥80	1	
< 120 / <80 (untreated)[f]	0.64 (0.47-0.86)	
**Cancer mortality[$]**		
**BMI, kg/m²**		NA
≥25	1	
<25	NA	
**Healthy diet score[¥]**		NA
< 2 components	1	
≥ 2 components	NA	
**Blood pressure, mm Hg**		NA
SBP ≥140 or DBP ≥90 mmHg	1	
SBP 120–139 or 80=<DBP <90 mmHg or treated to goa	NA	
<120/80 mmHg (untreated)[f]	NA	

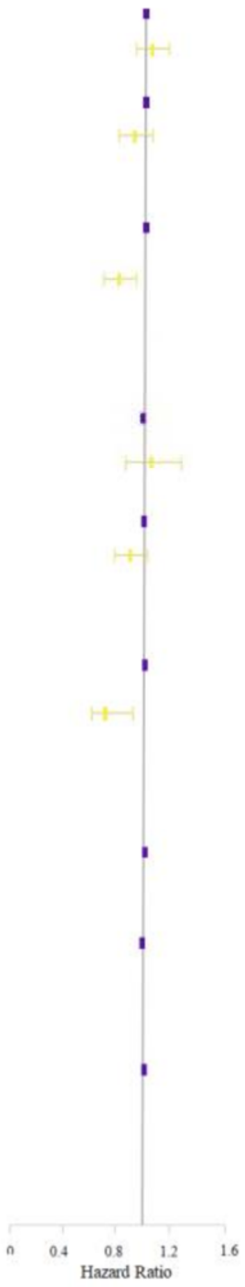

© 2019 Han L et al. *JAMA Network Open.*

Abbreviations:BMI-WHR, body mass index-waist-hip ratio; NHANES, National Health and Nutrition Examination Survey. The previous paper refer to Yang's et al., JAMA 2012 analysis for AHA-recommended LS7 metrics[3].

*. The classification of BMI combined with WHR was based on the reference 2 in the paper. BMI-WHR category 3 (normal weight with central obesity) was defined as "poor".

†. The healthy diet score was calculated based on the healthy eating index advocated by Dietary Guidelines for Americans 2015-2020 (Eighth Edition). The tertiles of HEI was classified as ideal, intermediate and poor, respectively.

‡. Adjusted for age, sex, race/ethnicity, educational attainment, alcohol intake, smoking status, physical activity, BMI, WHR, healthy diet score, total cholesterol level, systolic BP, diastolic BP, and HbA1c value as appropriate. For additional adjustment, when we investigate the association between each revised LS7 metric and outcomes, the relevant variables were excluded, for example, when the association between goal levels of untreated BP and outcomes was analyzed, systolic BP and diastolic BP were not included in the model.

§. Adjusted for age and sex.

‖. Adjusted for age, sex, race/ethnicity, educational attainment, alcohol intake, family history of cardiovascular disease, smoking status, physical activity, body mass index, health diet score, total cholesterol level, blood pressure, and HbA_{1c} value.

#. Adjusted for age, sex, race/ethnicity, educational attainment, alcohol intake, family history of CVD, smoking status, physical activity, BMI, healthy diet score, total cholesterol level, blood pressure, and HbA_{1c} value.

¥. The first dietary metrics include Fruits and vegetables⩾4.5 cups per day, Fish⩾two 3.5-oz servings per week (preferably oily fish), Fiber-rich whole grains (⩾1.1 g of fiber per 10 g of carbohydrate) ⩾three 1-oz-equivalent servings per day, Sodium: <1500 mg per day, Sugar-sweetened beverages⩽450 kcal (36 oz) per week; The secondary dietary metrics include Nuts, legumes, and seeds ⩾4 servings per week; Processed meats: none or ⩽2 servings per week, Saturated fat <7% of total energy intake.

$. The results for the AHA-recommended LS7 metrics and cancer mortality were not available.

© 2019 Han L et al. *JAMA Network Open.*

eFigure 3: The interaction between age, education and LS7 health metrics on mortality outcomes

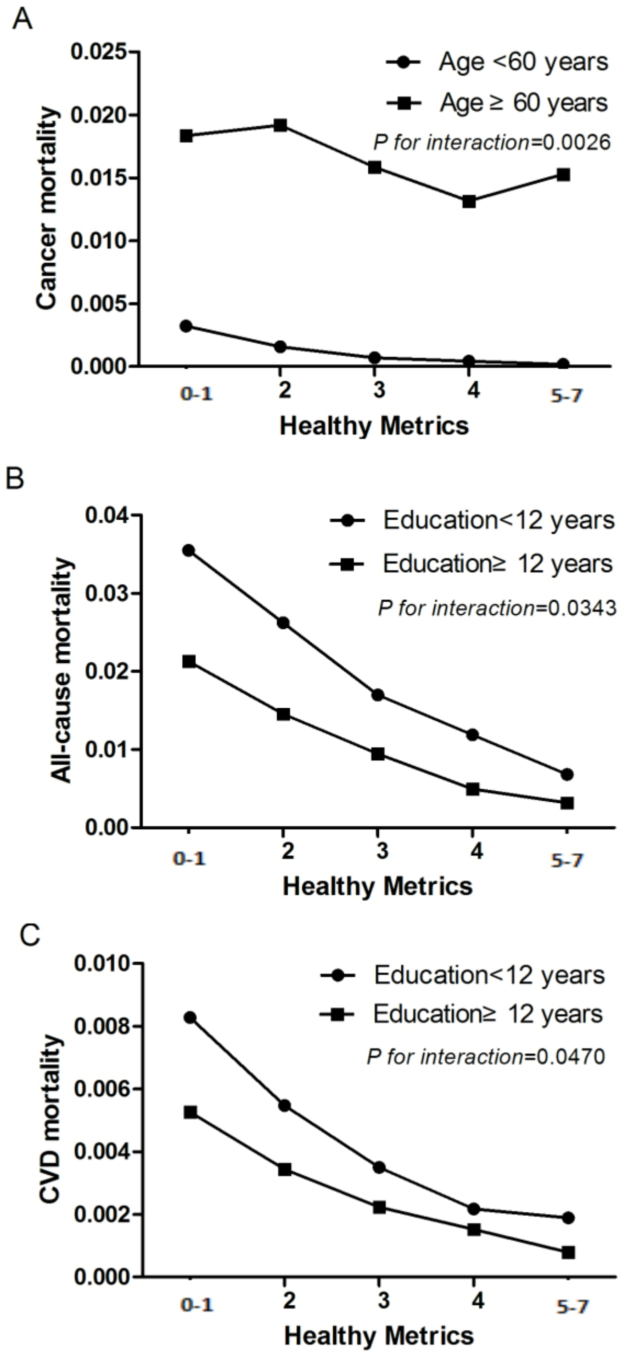

© 2019 Han L et al. *JAMA Network Open.*
